# Development and evaluation of an algorithm to link mothers and infants in two US commercial healthcare claims databases for pharmacoepidemiology research

**DOI:** 10.1186/s12874-023-02073-6

**Published:** 2023-10-21

**Authors:** James Weaver, Jill H. Hardin, Clair Blacketer, Alexis A. Krumme, Melanie H. Jacobson, Patrick B. Ryan

**Affiliations:** Janssen Research & Development, 1125 Trenton-Harbourton Rd, Titusville, NJ 08560 USA

**Keywords:** Data linkage, Pharmacoepidemiology, Drug safety, Perinatal research, Real-world databases

## Abstract

**Background:**

Administrative healthcare claims databases are used in drug safety research but are limited for investigating the impacts of prenatal exposures on neonatal and pediatric outcomes without mother-infant pair identification. Further, existing algorithms are not transportable across data sources. We developed a transportable mother-infant linkage algorithm and evaluated it in two, large US commercially insured populations.

**Methods:**

We used two US commercial health insurance claims databases during the years 2000 to 2021. Mother-infant links were constructed where persons of female sex 12–55 years of age with a pregnancy episode ending in live birth were associated with a person who was 0 years of age at database entry, who shared a common insurance plan ID, had overlapping insurance coverage time, and whose date of birth was within ± 60-days of the mother’s pregnancy episode live birth date. We compared the characteristics of linked vs. non-linked mothers and infants to assess similarity.

**Results:**

The algorithm linked 3,477,960 mothers to 4,160,284 infants in the two databases. Linked mothers and linked infants comprised 73.6% of all mothers and 49.1% of all infants, respectively. 94.9% of linked infants’ dates of birth were within ± 30-days of the associated mother’s pregnancy episode end dates. Characteristics were largely similar in linked vs. non-linked mothers and infants. Differences included that linked mothers were older, had longer pregnancy episodes, and had greater post-pregnancy observation time than mothers with live births who were not linked. Linked infants had less observation time and greater healthcare utilization than non-linked infants.

**Conclusions:**

We developed a mother-infant linkage algorithm and applied it to two US commercial healthcare claims databases that achieved a high linkage proportion and demonstrated that linked and non-linked mother and infant cohorts were similar. Transparent, reusable algorithms applied to large databases enable large-scale research on exposures during pregnancy and pediatric outcomes with relevance to drug safety. These features suggest studies using this algorithm can produce valid and generalizable evidence to inform clinical, policy, and regulatory decisions.

**Supplementary Information:**

The online version contains supplementary material available at 10.1186/s12874-023-02073-6.

## Background

Pregnancy is characterized by distinct periods of embryonic development representing critical exposure windows for children’s health [1]. Exposures before or during pregnancy, including pharmaceuticals, can affect conception, fetal development, pregnancy outcomes, and children’s health. While up to 90% of women take medication during pregnancy [2, 3], drug safety evidence is scarce because clinical trials often exclude pregnant people [4–6]. Mechanisms for generating pregnancy drug safety evidence are available, such as teratology information services [7], pregnancy and birth registries [8–12], case control studies [13], prospective cohort studies [14], and linked registry and prescription data resources [15]. However, these approaches often lack power to adequately assess rare exposures or outcomes, suffer from information biases, are slow to deliver results, may reflect selected populations, and are resource intensive. This research landscape produces an incomplete understanding of the benefits and risks of prenatal medication use and resultant birth outcomes. Timely and robust evidence is urgently needed in this population, as highlighted by the COVID-19 pandemic and the lack of efficacy and safety data for vaccine receipt during pregnancy.

Calls have been made to use real-world data (RWD) to study medication effects in pregnancy and are increasingly accepted by health authorities as part of post-authorization safety commitments [16, 17]. Large, administrative healthcare databases for pregnancy research are advantageous because they include large samples, multi-therapeutic area drug dispensing and diagnosis reimbursement claims, longitudinal patient observation, and reflect routine-care clinical practice [18].

To assess prenatal exposures on infant outcomes in RWD requires implementing algorithms to define pregnancy episodes and to link live births to infant records, which is challenging in the United States where national health record identifiers are absent. Mother-infant linkage has been conducted using US administrative healthcare databases, including among Medicaid, commercially-insured, and Military Health System populations [19–24]. Other efforts, such as the Medication Exposure in Pregnancy Risk Evaluation Program (MEPREP) [25, 26], have linked administrative and electronic health record data to state birth records. However, details on linkage confidence and evaluation are sparse [27].

Our study builds on past efforts to create mother-infant linked cohorts in RWD. The objective of this work was to link mother and infant data using two large, US commercial insurance databases. We also sought to evaluate the algorithm through comprehensive characterization comparisons between linked and non-linked mothers and infants. In contrast to other linkage studies that use proprietary algorithms, our algorithm is publicly available. The algorithm was developed for use against the Observational Medical Outcomes Partnership (OMOP) Common Data Model (CDM) [28, 29], so it may be applicable to similar databases that have been standardized. Our linkage algorithm furthers earlier linkage work based on insurance enrollment ID matching only, by applying additional temporal criteria intended to increase linkage confidence.

## Methods

### Data sources

The study used two health insurance claims databases, IBM® Marketscan® Commercial Database (CCAE)[2000–2022] and Optum’s de-identified Clinformatics® Data Mart Database (Clinformatics®)[2000–2021]. Both contain de-identified, patient-level, encounter-based, longitudinal, employer-based US administrative health insurance claims records and include inpatient and outpatient diagnoses, procedures, and outpatient prescription dispensing records. Both databases use a unique insurance enrollment ID for identifying beneficiaries and their dependents under a single, primary insurance holder account. Both databases were transformed to the OMOP CDM, which provides a standardized representation of database structure and clinical content [30] to enable consistent analysis across disparate healthcare databases [31, 32]. Detailed database descriptions are in Additional file 1.

### Linkage algorithm

The linkage algorithm relies on and is distinct from an algorithm for identifying pregnancy episodes and outcomes [33]. The pregnancy episodes algorithm was previously described, implemented, and validated in several administrative healthcare databases, including those utilized in this study [33]. In the pregnancy episodes algorithm, pregnancy outcomes (live births, stillbirths, abortions, and ectopic pregnancies) with associated dates were identified among women aged 12–55 years. Second, it estimated pregnancy start dates using a hierarchy of pregnancy markers, such as last menstrual period, amenorrhea, urine tests, and ultrasounds. The algorithm was validated through clinical adjudication of 700 electronic pregnancy episode profiles from Clinformatics® and the Clinical Practice Research Database that demonstrated high agreement between algorithm results and reviewers on 6 operating characteristics. This algorithm is currently being updated to include gestational age indicators in the ICD-10-CM vocabulary [34, 35].

#### Step 1: identify candidate mothers and infants

We first identified candidate mothers as females whose pregnancy episode(s) ended with live birth and occurred during a period of insurance enrollment.

Multiple periods of insurance enrollment were combined into a single observation period provided gaps between an enrollment period end and subsequent start date were ≤ 30 days. We identified candidate infants as persons whose year of birth was the same as their first observation period start year (i.e., were 0 years of age at observation period start) and had an insurance enrollment ID shared with a candidate mother. Candidate infants’ date of birth (DOB) was set as year, month, and day. Year of birth was available for all persons in both databases. Month and day were unavailable in the data sources we used through the patient de-identification process, so we inferred these components from observation period start month and day. Most day of birth values were set as 1 because insurance enrollment typically begins on the first day of a month. We refer to this date as the inferred date of birth, rather than the true date of birth, which we assert is the delivery date of the corresponding linked mother, where links were established. The algorithm will use month and day of birth if available but will set these values to month and day of enrollment start otherwise. This supports algorithm transportability if used in other insurance claims databases where birth date information may or may not be redacted.

#### Step 2: identify candidate mother-infant links

We identified candidate links between mothers and infants where they matched on insurance enrollment ID and the candidate infant’s inferred DOB occurred during a candidate mother’s observation period.

#### Step 3: classify probable mother-infant links

We identified probable links between mothers and infants by restricting to those where the candidate infant’s DOB occurred within ± 60 days of the candidate mother’s pregnancy episode end date. This correspondence window was varied in a sensitivity analysis (Additional file 1).

#### Step 4: exclude ambiguous mother-infant links

In Step 2, we identified rare instances where multiple mothers could be associated with a single infant. These records were excluded from analysis.

### Cohorts used in algorithm evaluation

Nine cohorts were constructed to compare characteristics between linked vs. non-linked mothers and infants. The index date refers to the temporal reference against which covariates were constructed.

1. Mothers linked to ≥ 1 infant indexed at pregnancy episode start.
2. Mothers linked to ≥ 1 infant indexed at pregnancy episode end.
3. Infants linked to a mother indexed at inferred DOB.
4. Mothers not linked to an infant indexed at pregnancy episode start.
5. Mothers not linked to an infant indexed at pregnancy episode end.
6. Infants not linked to a mother indexed at inferred DOB.
7. Candidate mothers indexed at pregnancy episode start.
8. Candidate mothers indexed at pregnancy episode end.
9. Candidate Infants indexed at inferred DOB.

Note that cohorts 7, 8, and 9 were constructed to create cohorts 4, 5, and 6. For example, cohort 4 equals mothers in cohort 7 with mothers from cohort 1 removed. Cohorts 1–3 and 4–6 were used in characteristic comparisons.

### Characterization analyses

We characterized mother cohorts using demographic, clinical, and healthcare utilization covariates relative to each index date: once with covariates that reflect events observed during the year before or on the pregnancy episode start date (reported in Table 1), and again with covariates that reflect events observed during the year before or on the delivery date (reported in Table 2). The intent of Table 1 is to describe pre-pregnancy characteristics, whereas the intent of Table 2 is to describe characteristics that occur mostly during pregnancy (recognizing the limitation that approximately 3 months of the one-year covariate construction window is before pregnancy start). We characterized the infant cohorts with covariates that reflect events observed on or during the year after the inferred DOB. See Additional file 1 for details on how demographic, clinical, and healthcare utilization covariates were measured. For example, if a procedure code for a basic metabolic panel was observed on a patient record 3 months before delivery date, a measurement covariate would be constructed indicating that the test was performed but it would not include any lab results.

**Table 1:** Selected characteristics of linked and non-linked mothers, measured 365 days before and including pregnancy start

	CCAE			Clinformatics®		
Characteristic	Linked % (n = 2,528,482)	Non-linked % (n = 995,892)	SMD	Linked % (n = 1,589,010)	Non-linked % (n = 420,199)	SMD
Index year						
2000	0.61	0.72	0.013	1.35	1.39	0.003
2001	0.94	1.13	0.019	3.83	3.77	-0.003
2002	1.92	2.04	0.009	4.71	4.54	-0.008
2003	3.11	2.64	-0.028	4.84	4.65	-0.009
2004	4.02	3.39	-0.033	4.8	4.49	-0.015
2005	3.97	3.27	-0.037	6.18	4.09	-0.095
2006	4.74	4.11	-0.031	6.3	4.76	-0.067
2007	4.93	4.36	-0.027	6.31	4.81	-0.065
2008	6.07	5.25	-0.036	5.93	4.64	-0.058
2009	6.17	5.83	-0.014	5.12	5.18	0.003
2010	7.45	6.47	-0.039	4.54	6.01	0.066
2011	8.16	8.36	0.007	4.37	6.45	0.092
2012	6.85	7.69	0.032	4.87	5.33	0.021
2013	6.96	7.26	0.012	4.71	4.81	0.005
2014	5.9	6.82	0.038	4.41	4.78	0.018
2015	5.34	5.99	0.028	4.6	5.02	0.02
2016	4.96	5.45	0.022	4.67	5.17	0.023
2017	4.87	5.11	0.011	4.81	5.28	0.022
2018	5.09	5.27	0.008	4.78	5.11	0.015
2019	4.5	4.79	0.014	4.44	4.89	0.021
2020	3.43	4.06	0.033	4.33	4.65	0.015
2021	0	0.01	0.008	0.11	0.18	0.017
Index month						
1	10	9.71	-0.01	9.05	9.14	0.003
2	7.58	10.71	0.109	7.79	8.32	0.019
3	6.1	14.36	0.275	7.86	9.19	0.048
4	7.4	8.38	0.037	7.42	7.7	0.011
5	8.48	7.07	-0.053	8.24	7.97	-0.01
6	8.42	6.8	-0.061	8.07	7.8	-0.01
7	8.88	6.89	-0.074	8.41	8.11	-0.011
8	8.79	6.84	-0.073	8.37	8.01	-0.013
9	8.7	7.1	-0.059	8.52	8.18	-0.012
10	8.73	7.43	-0.048	8.79	8.48	-0.011
11	8.49	7.23	-0.047	8.7	8.4	-0.011
12	8.43	7.47	-0.036	8.78	8.71	-0.003
Age (years)						
Mean	31.22	27.36	-0.483	30.93	27.94	-0.357
Std. deviation	4.68	6.48		4.88	6.81	
Median	31	27		31	28	
Prior observation time (days)						
Mean	737.21	833.74	0.079	715.62	778.33	0.054
Std. deviation	743.73	978.46		741.25	893.25	
Median	503	489		476	472	
Post observation time (days)						
Mean	1357.52	959.98	-0.265	1221.15	930.43	-0.212
Std. deviation	1193.69	905.81		1092.51	829.61	
Median	929	648		825	642	
Pregnancy episode length (days)						
Mean	273.15	269.83	-0.111	272.73	270.33	-0.08
Std. deviation	18.44	23.58		18.89	23.27	
Median	278	277		278	278	
Distinct conditions						
Mean	6.67	6.77	0.01	6.95	7.45	0.045
Std. deviation	6.73	7.34		7.38	8.18	
Median	5	4		5	5	
Distinct drug ingredients						
Mean	4.68	4.62	-0.009	4.14	4.44	0.04
Std. deviation	5.39	5.49		5.17	5.5	
Median	3	3		3	3	
Distinct procedures						
Mean	7.4	6.62	-0.075	7.08	6.92	-0.015
Std. deviation	7.39	7.28		7.61	7.71	
Median	5	5		5	5	
Distinct measurements						
Mean	6.7	6.29	-0.025	13.62	12.48	-0.03
Std. deviation	11.93	11.86		27.48	25.9	
Median	3	3		3	3	
Distinct visit types						
Outpatient Visit						
Mean	6.61	5.51	-0.085	6.38	5.89	-0.036
Std. deviation	9.81	8.31		10.47	8.98	
Median	4	3		3	3	
Inpatient Visit						
Mean	0.11	0.11	0	0.1	0.11	0.003
Std. deviation	1.11	1.25		0.77	0.94	
Median	0	0		0	0	
Emergency Room Visit						
Mean	0.16	0.34	0.09	0.38	0.33	-0.007
Std. deviation	1.16	1.56		6.14	4.89	
Median	0	0		0	0	
Charlson comorbidity index						
Mean	0.19	0.2	0.002	0.22	0.24	0.017
Std. deviation	0.85	0.89		0.93	1.02	
Median	0	0		0	0	

CCAE: IBM® Marketscan® Commercial Database; Clinformatics®: Optum’s de-identified Clinformatics® Data Mart Database; SMD: Standardized difference of means

**Table 2:** Selected characteristics of linked and non-linked mothers, measured 365 days before and including pregnancy end

	CCAE			Clinformatics®		
Characteristic	Linked % (n = 2,528,482)	Non-linked % (n = 995,892)	SMD	Linked % (n = 1,589,010)	Non-linked % (n = 420,199)	SMD
Index year						
2000	0.1	0.31	0.047	0.01	0.02	0.01
2001	0.64	0.87	0.026	2.01	2.2	0.013
2002	1.14	1.51	0.032	4.27	4.29	0.001
2003	2.21	2.26	0.003	4.82	4.67	-0.007
2004	3.32	2.87	-0.026	4.78	4.57	-0.01
2005	4.08	3.44	-0.034	5.07	4.05	-0.049
2006	4.03	3.37	-0.035	6.37	4.42	-0.087
2007	4.81	4.21	-0.029	6.3	4.84	-0.064
2008	5.26	4.71	-0.025	6.26	4.79	-0.064
2009	6.38	5.4	-0.042	5.81	4.58	-0.055
2010	6.17	6.26	0.004	4.8	5.62	0.037
2011	7.65	6.81	-0.033	4.49	6.14	0.074
2012	8.04	8.94	0.032	4.4	6.46	0.091
2013	6.7	6.97	0.01	5.07	4.9	-0.008
2014	6.97	7.38	0.016	4.56	4.74	0.009
2015	5.45	6.15	0.03	4.47	4.86	0.018
2016	5.44	6.03	0.025	4.73	5.12	0.018
2017	4.95	5.25	0.014	4.68	5.2	0.024
2018	4.91	5.18	0.012	4.8	5.23	0.02
2019	4.97	5.12	0.007	4.74	5.1	0.017
2020	4.43	4.68	0.012	4.34	4.76	0.021
2021	2.33	2.28	-0.003	3.23	3.43	0.011
Index month						
1	8.08	6.7	-0.053	7.55	7.44	-0.004
2	7.77	6.19	-0.062	7.43	7.04	-0.015
3	8.74	6.84	-0.071	8.32	8.01	-0.011
4	8.56	6.65	-0.072	8.14	7.83	-0.012
5	8.97	6.9	-0.077	8.58	8.13	-0.016
6	8.7	7.2	-0.055	8.47	8.19	-0.01
7	8.71	7.48	-0.045	8.76	8.51	-0.009
8	8.73	7.46	-0.047	8.96	8.74	-0.008
9	8.52	7.6	-0.034	8.71	8.63	-0.003
10	9.68	8.96	-0.025	8.77	8.85	0.003
11	8.37	10.81	0.083	8.27	8.64	0.013
12	5.17	17.22	0.389	8.04	9.99	0.068
Age (years)						
Mean	31.99	27.98	-0.504	31.68	28.66	-0.36
Std. deviation	4.67	6.44		4.87	6.81	
Median	32	27		32	28	
Prior observation time (days)						
Mean	1010.36	1103.57	0.076	988.36	1048.67	0.052
Std. deviation	743.4	978.27		741	893.1	
Median	777	758		749	743	
Post observation time (days)						
Mean	1084.37	690.15	-0.263	948.41	660.09	-0.21
Std. deviation	1192.66	905.33		1091.46	828.86	
Median	656	377		552	371	
Distinct conditions						
Mean	19.81	20.75	0.061	20.21	21.43	0.076
Std. deviation	10.21	11.4		10.64	11.96	
Median	18	18		18	19	
Distinct drug ingredients						
Mean	5.3	5.65	0.045	5.4	6	0.074
Std. deviation	5.35	5.61		5.39	5.84	
Median	4	4		4	4	
Distinct procedures						
Mean	17.46	16.82	-0.054	18.6	18.55	-0.004
Std. deviation	8.44	8.51		8.72	9.11	
Median	16	15		18	17	
Distinct measurements						
Mean	20.85	20.95	0.004	44.01	41.65	-0.046
Std. deviation	15.57	16.57		36.44	36.03	
Median	18	18		32	27	
Distinct visit types						
Outpatient Visit						
Mean	15.53	14.21	-0.089	15.7	14.76	-0.054
Std. deviation	11.33	9.52		13.44	10.96	
Median	13	12		12	12	
Inpatient Visit						
Mean	1.13	1.14	0.006	1.14	1.12	-0.019
Std. deviation	0.67	0.69		0.71	0.64	
Median	1	1		1	1	
Emergency Room Visit						
Mean	0.33	0.67	0.146	1.03	0.98	-0.007
Std. deviation	1.36	1.85		5.63	4.52	
Median	0	0		0	0	
Charlson comorbidity index						
Mean	0.3	0.3	0.001	0.32	0.34	0.016
Std. deviation	0.9	0.94		0.98	1.08	
Median	0	0		0	0	

CCAE: IBM Commercial Database; Clinformatics®: Optum’s de-identified Clinformatics® Data Mart Database; SMD: Standardized difference of means

Lastly, we compared characteristics between linked vs. non-linked mothers and infants to evaluate differences between populations that did and did not meet linkage algorithm criteria. We made covariate comparisons by calculating the standardized mean difference (SMD) for each covariate in units of the pooled standard deviation, a metric uninfluenced by large sample sizes [36], and interpreted SMD values > 0.1 as meaningfully different [37, 38].

## Results

All source code and an interactive web application for viewing full results is available at https://data.ohdsi.org/MotherInfantLinkEval/. A reader can navigate to this web-based application to review the full characterization results set for each linked vs. non-linked comparison. By default, the table reports characteristic prevalence results for linked vs. non-linked cohorts sorted by largest to smallest standardized mean difference between characteristic prevalence. Additionally, a reader can search for characteristics of interest using the search bar.

**Fig. 1:**
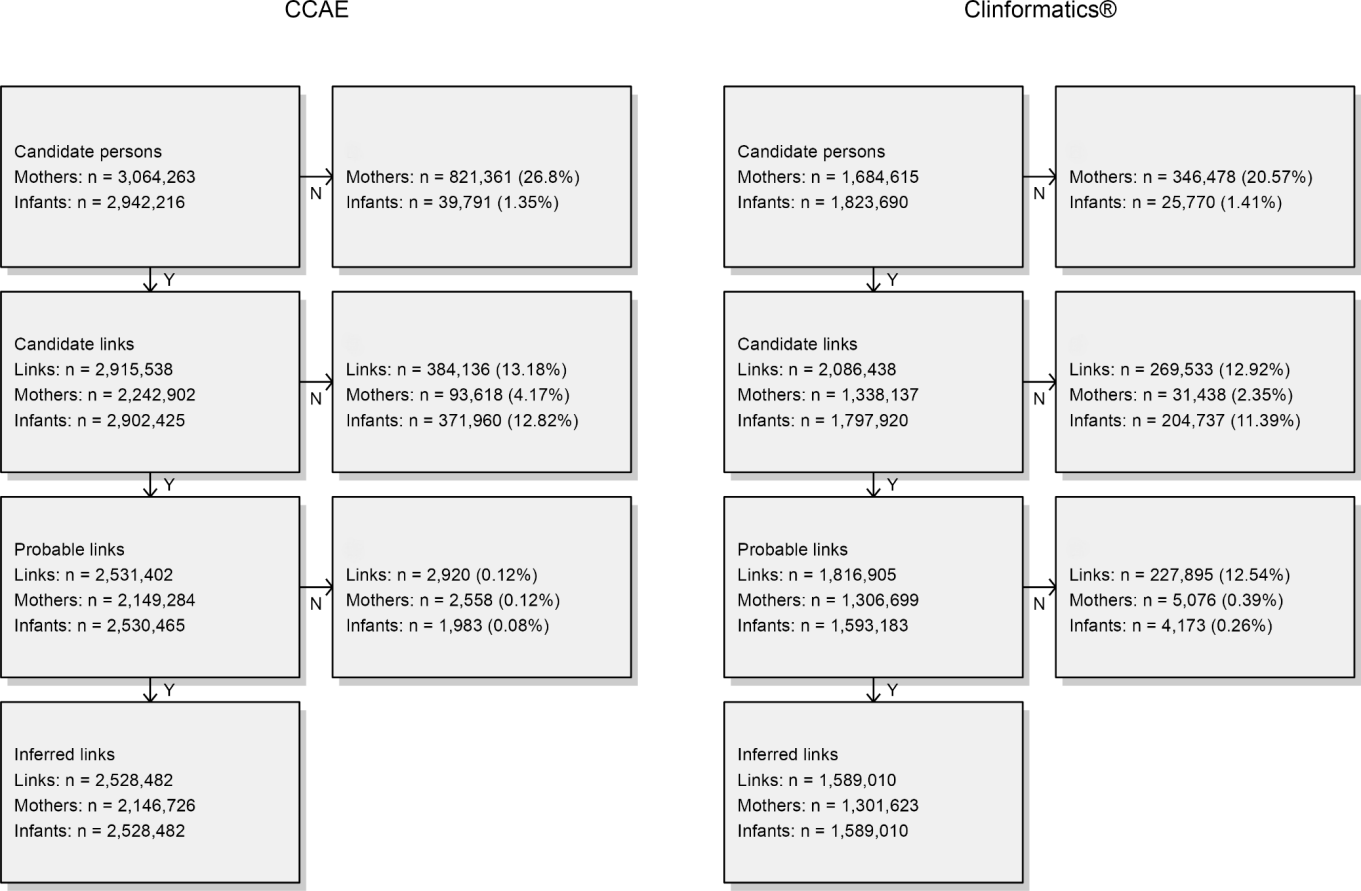
Mother-infant linkage algorithm attrition diagram **Panel A**: IBM® Marketscan® Commercial Database **Panel B**: Optum de-identified Clinformatics® Data Mart Database Footnote: Candidate mothers: women whose pregnancy episode(s) ended with live birth and occurred during a mother’s observation period; Candidate infants: persons who were 0 years of age at observation period start; Candidate links: mothers-infant pairs who matched on insurance enrollment ID infant’s date-of-birth occurred during a candidate mother’s observation period; Probable links: candidate links where candidate infants date-of-birth occurred within ± 60 days of the candidate mother’s pregnancy episode end date; Inferred links: removal of probable links where multiple mothers associated with one infant

Figure 1 depicts step-by-step attrition of the linkage algorithm.

In CCAE, 3,064,263 candidate mothers and 2,942,216 candidate infants were identified in Step 1, of whom 26.8% and 1.4% were dropped respectively during Step 2, resulting in 2,915,538 candidate links. Links were reduced by 13.2% and 0.1% in steps 3 and 4 respectively, which resulted in 2,528,482 links: 2,146,726 linked mothers, and 2,528,482 linked infants. 31.3% of linked infant’s DOB were on the same day as their linked mother’s pregnancy episode end date and 58.3%, 71.5%, and 92.1% occurred within ± 7 days, ± 14 days, and ± 30 days, respectively. Linked infant’s DOB was on average 5.9 days (SD = 15.1, median = 1) after the pregnancy episode end date. Linked mothers comprised 70.1% of all mothers (n = 3,064,263) and linked infants comprised 51.2% of all infants (n = 4,935,376) (Additional file 1).

In Clinformatics®, 1,684,615 candidate mothers and 1,823,690 candidate infants were identified, of whom 20.6% and 1.4% were dropped respectively during Step 2, resulting in 2,086,438 candidate links. Links were reduced by 12.9% and 12.5% in steps 3 and 4 respectively, which resulted in 1,589,010 links: 1,301,623 linked mothers and 1,589,010 linked infants. 67.4% of linked infant’s DOB were on the same day as their linked mother’s pregnancy episode end date and 98.0% 98.6%, and 99.3% occurred within ± 7 days, ± 14 days, and ± 30 days, respectively. Linked infants’ DOB was on average 0.7 days (SD = 4.0, median = 0) after the pregnancy episode end date. Linked mothers comprised 77.3% of all mothers (n = 1,684,615) and linked infants comprised 47.0% of all infants (n = 3,379,811)(Additional file 1).

**Fig. 2:**
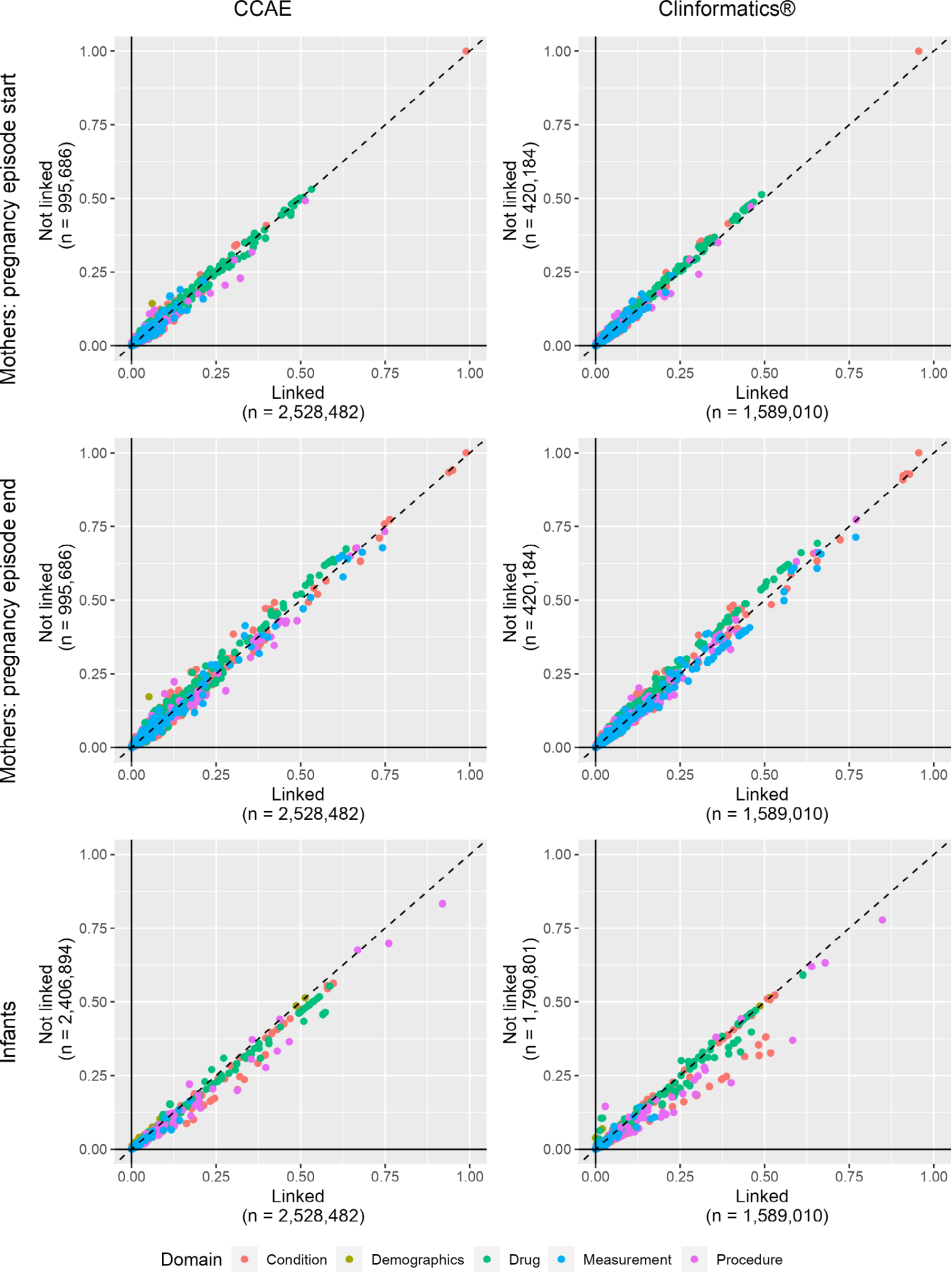
Demographic, drug exposure, condition, procedure, measurement, and visit occurrence prevalence Footnote: The x-axes display the prevalence of each covariate in the linked populations and the y-axes display the prevalence of each covariate in the non-linked populations. Data points that lay on the diagonal represent covariates that are equally prevalent in the linked and non-linked populations. Data points to the right the diagonal represent covariates that are more prevalent in the linked populations and those to the left are more prevalent in the non-linked populations

Figure 2 depicts the comparative prevalence of demographic, drug exposure, condition, procedure, and measurement occurrence covariates for the linked vs. non-linked mother and infant cohorts.

The plots illustrate that the characteristics of linked and non-linked mothers were generally similar. However, infant characteristics, including conditions, measurements, drugs, and procedures were more prevalent among linked vs. non-linked infants. Large SMD covariates with greater prevalence among the linked infants included procedural billing records related to infant care, infant screening procedures, immunizations, and some conditions (see web application to review all characteristics). We also observed a greater prevalence of birth-related covariates among linked infants than non-linked infants (e.g., “Single live birth”, ”Finding related to pregnancy”). Despite these differences, we still observed absolute SMDs of < 0.1 for > 99% of covariates across all algorithm implementations of each linked vs. non-linked comparison in both databases where the number of covariate comparisons ranged from 58,611 (CCAE infants) to 68,368 (Clinformatics® mothers pregnancy end).

Table 1 reports characteristics and SMDs of linked vs. non-linked mothers for several characteristics measured relative to pregnancy episode start date. Pregnancy episode starts were equally distributed by year over the study period, although index dates in non-linked mothers were more common in February and March in CCAE. Mean age was greater among linked mothers in both databases (CCAE: 31.2 vs. 27.4 years, Clinformatics®: 30.9 vs. 27.9 years). There was greater post-pregnancy mean observation time among linked mothers in both databases (CCAE: 1358 vs. 960 days, Clinformatics®: 1221 vs. 930 days) and mean pregnancy episode length was greater among linked mothers in CCAE (273 vs. 270 days). Linked vs. non-linked mothers did not differ in clinical event counts, healthcare utilization, and Charlson comorbidity index in either database.

Table 2 reports characteristics and SMDs of linked vs. non-linked mothers for the same characteristics as Table 1 except for pregnancy episode length but were measured relative to pregnancy episode end date. Age was greater among linked mothers in CCAE (32.0 vs. 30.9 years), which reflects the slightly greater linked pregnancy episode lengths reported above. There was greater post-pregnancy observation time among linked mothers in both databases (CCAE: 1084 vs. 690 days, Clinformatics®: 948 vs. 660 days). Although uncommon, emergency room visits were greater among non-linked mothers in CCAE (0.7 vs. 0.3).

Table 3 reports characteristics and SMDs of linked vs. non-linked infants for several characteristics measured at their inferred birth dates (enrollment start date). Non-linked births were more common in the early study period (2000–2003) in both databases. There was greater average post-birth observation time among linked infants in both databases (CCAE: 1060 vs. 886 days., Clinformatics®: 855 vs. 751 days). Average condition (CCAE: 6.8 vs. 5.7, Clinformatics®: 7.8 vs. 6.4) and procedure (CCAE: 11.6 vs. 9.9, Clinformatics®: 12.3 vs. 10.0) occurrences were greater among linked infants. Healthcare utilization (i.e., outpatient and inpatient visits) was similarly greater among linked infants.

**Table 3:** Selected characteristics and standardized differences of linked and non-linked infants

	CCAE			Clinformatics®		
Characteristic	Linked % (n = 2,528,482)	Non-linked % (n = 2,406,894)	SMD	Linked % (n = 1,589,010)	Non-linked % (n = 1,790,801)	SMD
Sex						
gender = FEMALE	48.71	48.66	-0.001	48.57	48.55	0
gender = MALE	51.29	51.34	0.001	51.43	51.45	0
Index year						
2000	0.09	1.08	0.13	0.01	3.87	0.283
2001	0.64	1.07	0.046	2.01	6.89	0.238
2002	1.14	2.54	0.104	4.27	4.93	0.032
2003	2.21	3.95	0.101	4.82	4.66	-0.008
2004	3.32	4.3	0.051	4.78	4.49	-0.014
2005	4.08	4.44	0.018	5.07	6.65	0.067
2006	4.03	4.99	0.047	6.37	6.06	-0.013
2007	4.81	4.8	0	6.3	5.97	-0.014
2008	5.26	6.23	0.042	6.26	5.06	-0.052
2009	6.38	6.57	0.008	5.81	4.13	-0.077
2010	6.17	7.5	0.053	4.79	3.56	-0.062
2011	7.65	7.35	-0.011	4.49	3.52	-0.05
2012	8.04	7.25	-0.03	4.39	3.51	-0.045
2013	6.7	6.32	-0.015	5.08	3.99	-0.052
2014	6.98	6.29	-0.028	4.56	4.02	-0.026
2015	5.45	4.15	-0.061	4.47	4.68	0.01
2016	5.44	4.1	-0.063	4.73	4.84	0.005
2017	4.95	4.02	-0.045	4.68	4.68	0
2018	4.91	4.47	-0.021	4.8	4.44	-0.017
2019	4.97	3.65	-0.065	4.74	4.04	-0.034
2020	4.43	3.43	-0.051	4.34	3.49	-0.044
2021	2.34	1.49	-0.062	3.24	2.54	-0.042
Index month						
1	4.78	4.57	-0.01	7.42	6.15	-0.05
2	7.11	6.37	-0.03	7.39	5.91	-0.059
3	8.24	7.42	-0.031	8.3	6.73	-0.06
4	8.8	8.49	-0.011	8.15	6.97	-0.045
5	8.67	8.58	-0.003	8.54	10.15	0.056
6	8.64	9.04	0.014	8.47	8.5	0.001
7	9	10.52	0.052	8.79	9.93	0.039
8	8.53	9.88	0.047	8.93	9.57	0.022
9	8.42	10.31	0.065	8.74	9.95	0.041
10	9.66	9.67	0	8.78	9.38	0.021
11	9.32	7.88	-0.051	8.3	8.32	0.001
12	8.84	7.28	-0.057	8.19	8.44	0.009
Post observation time (days)*						
Mean	1060.01	886.47	-0.106	855.02	751.11	-0.091
Std. deviation	1206.57	1113.97		1103.29	985.63	
Median	618	486		431	395	
Distinct conditions						
Mean	6.81	5.73	-0.115	7.75	6.39	-0.13
Std. deviation	6.9	6.39		7.64	7.11	
Median	5	4		6	5	
Distinct drug ingredients						
Mean	2.69	2.45	-0.049	8.55	7.44	-0.066
Std. deviation	3.43	3.38		12.05	11.76	
Median	1	1		2	2	
Procedures						
Mean	11.55	9.87	-0.164	12.28	9.99	-0.213
Std. deviation	7.42	7		7.83	7.36	
Median	10	9		11	9	
Distinct measurements						
Mean	2.63	2.23	-0.056	3.29	2.83	-0.044
Std. deviation	4.96	4.92		7.43	7.61	
Median	1	1		1	1	
Distinct visit types						
Outpatient Visit						
Mean	10.09	8.65	-0.155	9.47	8.32	-0.107
Std. deviation	6.65	6.46		7.74	7.51	
Median	9	8		9	7	
Inpatient Visit						
Mean	0.67	0.5	-0.236	1.03	0.69	-0.494
Std. deviation	0.51	0.57		0.47	0.51	
Median	1	0		1	1	
Emergency Room Visit						
Mean	0.22	0.24	0.016	0.1	0.13	0.006
Std. deviation	0.84	0.91		3.95	3.45	
Median	0	0		0	0	

CCAE: IBM Commercial Database; Clinformatics®: Optum’s de-identified Clinformatics® Data Mart Database; SMD.: Standardized difference of means

*Post-index observation time was measured from the linked mother’s delivery date (true birth date) to end of observation time

The final person and record counts for each of the 9 cohorts constructed by the 3 linkage algorithm implementations in each database are reported in Additional file 1. Result sets for the two algorithm sensitivity implementations are reported in Additional file 1. We observed similar stepwise attrition proportions across sensitivity implementations. Attrition proportions in the first births sensitivity implementation were greater in Step 3 because this is where first birth restrictions were made. There were no appreciable differences in linked vs. non-linked mother and infant characteristics across algorithm sensitivity implementations.

## Discussion

We developed and implemented an algorithm to infer mother-infant links in two large US commercial healthcare databases that exhibited high linkage coverage and similar characteristics across linked vs. non-linked persons. This signifies generalizability of linked mother-infant pairs to commercially insured source populations, which facilitates large-scale research on prenatal exposures and infant outcomes. This constitutes novel research by virtue of our emphasis on linked vs. non-liked characterization comparisons to support generalizability. Similarity of measured characteristics in linked vs. non-linked mother and infant records is supporting evidence that results produced by analyzing linked cohorts will generalize to the underlying source population, in this case commercially insured pregnant people and their infants. Our assessment of average linked-infant follow-up time (Clinformatics®: 855 days, CCAE: 1060 days) allows their inclusion in perinatal-exposure studies where outcomes of interested are not birth outcomes per se but longer-term infant conditions. Further, our linkage algorithm was implemented in the OMOP CDM, and the source code is publicly available. The utility of using standardized analytic routines against a standard data representation allows for transportable, complex algorithms to be implemented in other claims databases formatted to the OMOP CDM with no loss of fidelity [39].

Our algorithm identified > 3.4 million linked mothers and > 4.1 million linked infants. Access to large, linked populations makes feasible the study of a wide range of prescription drug exposures, maternal and neonatal outcomes, and subgroups that are often unavailable in smaller linked populations [40, 41] and registries [18, 42, 43]. This approach requires fewer study resources compared to studies that require primary data collection [44].

Across databases, linked mothers comprised 73.6% of all mothers with live births. In Clinformatics®, 77.3% of mothers were successfully linked to infants, which is lower but comparable to the 84% reported in a recent study using data from the same source with fewer linkage restrictions [19]. Despite similar methods, other linkage studies have reported mixed linkage coverage, suggesting that differences are due to data accuracy and/or availability variation across sources. Palmsten et al. linked Medicaid-enrolled mothers and infants and reported linkage coverage of 55.6% for inpatient deliveries, although with considerable variation by state (0–96%) [23], which the authors attributed to varying family identifier quality and use. A study in TRICARE enrollees in the Military Health System reported 90% of pregnancies ending in live births were linked with infants [24], which may be attributable to lower insurance coverage churn.

In our study, linked infants comprised 49.1% of all infants defined as persons 0 years of age at their observation period start. Contextualizing our linked infant coverage is difficult because most studies only report the proportion of linked pregnancies [19, 23]. However, Garbe et al. conducted a study using the German Pharmacoepidemiological Research Database (GePaRD), a claims database from four statutory health insurance providers, and reported that 77.3% of newborns were linked with mothers [45]. Additionally, a study among Medicaid enrollees in Tennessee reported 97% of infants were linked with a delivery, however such high coverage is likely explained by the use of vital record data with identifying information [41].

While our primary analysis used a ± 60-day window between infant DOB and mothers’ pregnancy episode end to identify candidate links, in sensitivity analyses, we observed high correspondence at 7, 14, and 30 days, including same-day correspondence of 31.3% in CCAE and 67.4% in Clinformatics®. Overall correspondence was greater in Clinformatics®, which may be due to more accurate and specific DOB information. Increasing the correspondence window to 90 days increased the proportion of linked infants by only 1.5% in CCAE and 0.2% in Clinformatics®, which we do not interpret as material because most of the correspondence occurred within ± 30 days.

Characteristic comparisons between linked and non-linked mothers revealed similar demographic, clinical, and healthcare utilization profiles. Our linkage evaluation largely supports the generalizability of the linked mother population, having compared thousands of covariates between linked and non-linked mother cohorts and observing few differences. Of note, two of the differences we found in both CCAE and Optum were also detected in a recent study using the Sentinel network: non-linked mothers were younger and had shorter gestations than linked mothers [46]. It is possible that these consistent differences are due to unmeasured factors associated with mother and infant not sharing the same insurance policy. Despite this, we found that SMDs were < 0.1 for nearly all observed characteristics, suggesting that in a prenatal exposure study on a small, exposed subset of the linked mother population, systematic differences between the study sample and non-linked mothers to whom the results will apply will be minimal.

Despite substantial similarity between linked and non-linked infants, we observed more differences than when comparing mothers. Of note, linked infants had greater total healthcare utilization and prevalence of individual clinical events, including birth and infant-care related claims. Because our algorithm linked mothers to infants by a shared insurance ID within a defined temporal interval, candidate infants whose inferred DOB fell outside of that interval would be non-linked and less likely to have their clinical events captured in the database. This suggests that some billing records may be attributed to family members on other insurance plans among the non-linked populations. Still, this evaluation supports the generalizability of the linked infant population. In cases of multiple pregnancies, linkages are made between maternal records and all live births from that pregnancy. In the event of a multifetal pregnancy ending in both a live birth and a stillbirth, codes associated with the still born infant may be observed on the live born infant record or on that of the linked mother. The occurrence of this scenario is expected to be very rare, with less than 0.5% of multifetal pregnancies experiencing a stillbirth in one study [47].

Despite CCAE and Clinformatics® both consisting of administrative claims data from large US commercial insurance plans, data content heterogeneity between them still exists, which could contribute to results differences between them. In Clinformatics®, we observed more situations where multiple women of child-bearing age were associated with one candidate infant. This suggests that more extended family members may be included on the same insurance plan in Clinformatics® than in CCAE, which would increase the situations where one infant is associated with > 1 candidate mother on the same health plan. Regarding selection bias, by excluding multiple women of child-bearing age on the same insurance plan, we may be selectively decreasing representation of large, varied families covered in the Clinformatics® database.

We note that several recent studies have established mother-infant linkage algorithms in claims databases with similar methods to the one described in this paper [23, 24, 34, 45, 48]. Specifically, linkage algorithms in the Clinformatics® [34] and in the CCAE [48] used infant dates of birth, maternal delivery dates, and family insurance IDs to link delivering mothers with infants. The algorithm used in CCAE captured slightly more links because it did not restrict to live births initially, had a wider correspondence window allowance, and when multiple mothers or pregnancies were associated with a single infant, it selected the earliest whereas our approach excluded those ambiguous links. While other studies have used related methods successfully [23, 24, 34, 45, 48], we show that our standardized approach works across multiple databases. The algorithm presented in this study offers a reproducible framework that can be implemented across different databases, particularly those transformed to the OMOP CDM. Further, we have characterized the populations of linked and unlinked mothers and children, which aids in contextualizing the output of these linkages and implications for their use in future research.

A strength of our study is the rigorous linkage approach utilizing insurance ID in addition to delivery and birth procedure dates in large US claims databases representing the commercially insured population. Further, we provide open-source code and a web-application to interactively review characterization results, which provides valuable context for the external validity of future studies among linked populations. Lastly, developing a reproducible mother-child linkage algorithm in large administrative databases facilitates evidence generation in pregnant populations with improved rigor by avoiding recall, referral, and self-selection biases inherent to registry or other primary data collection studies of prenatal medication use [18].

Using administrative healthcare claims databases in pharmacoepidemiologic research has limitations. Erroneously coded or missing diagnostic, procedure, and drug dispensing records results in misclassification which may under- or over-estimate exposures, covariates, health outcomes, other clinical events, and healthcare utilization [49]. Subsequent information bias that can result from misclassification is underappreciated [50] and could bias findings of future drug safety studies. Further, because the data do not provide exact date of birth information for non-linked infants, estimating event prevalence during 365-days post-birth is imprecise. This may result in misclassification by failing to capture events specifically related to the birth encounter itself. We observed these birth-related conditions and procedures as imbalanced in Fig. 2 and the infants tab of the web application.

Still, developing reliable mother-infant linkages in large healthcare databases has increased the capacity to examine associations between rare prenatal drug exposures and infant outcomes with sufficient power. For example, prenatal use of antidepressants, stimulants, antihypertensive medications, and sulfonamides have been studied in relation to validated congenital anomalies [51–55]. This has yielded needed real-world evidence on the safety of prenatal exposures.

While we found few differences between linked and non-linked populations suggestive of high internal validity to the underlying commercially insured US population, our results do not necessarily ensure external validity to those covered under other types of insurance or lacking coverage. The data in this study are representative of people with US-based, employer-sponsored health insurance, indicative of a higher socioeconomic status population. Given the established association between wealth and health [56, 57], care should be taken not to assume that linked vs. non-linked similarity we observed is consistent across other socioeconomic demographics. Further, administrative healthcare databases include detailed outpatient drug dispensing records but provide fewer details on inpatient dispensing records, prescriptions, or administrations typically available in electronic medical records. Additionally, we note that pregnancy episode length was slightly shorter in non-linked pregnancies (Table 1), but we do not believe this difference could substantially influence observed linked vs. non-linked maternal covariate differences in the year before birth, which were few. Lastly, our study has not been validated. However validation of a similar algorithm developed in claims data among Medicaid beneficiaries showed high positive predictive value [58].

## Conclusions

Our study reinforces the shift towards implementing pharmacoepidemiology studies on prenatal drug exposures utilizing large electronic healthcare data as a supplement to traditional pregnancy registries. Our algorithm and evaluation demonstrate the ability to assemble large mother-infant linked cohorts for investigating prenatal drug exposure effects on infant outcomes.

### Electronic supplementary material

Below is the link to the electronic supplementary material.

Supplementary Material 1

## Data Availability

The data that support the findings of this study are available from IBM® Marketscan® and Clinformatics® but restrictions apply to the availability of these data, which were used under license for the current study, and so are not publicly available. Aggregated (i.e. no person-level data) results that are the basis of the study findings are publicly available at: https://github.com/OHDSI/ShinyDeploy/tree/master/MotherInfantLinkEval/data.

## Abbreviations

MEPREP: Medication Exposure in Pregnancy Risk Evaluation Program
OMOP: Observational Medical Outcomes Partnership
CDM: Common Data Model
CCAE: IBM® Marketscan® Commercial Database
DOB: Date of birth
SMD: Standardized mean difference
GePaRD: German Pharmacoepidemiological Research Database
