# Supplementary material for: Development and evaluation of an algorithm to link mothers and infants in two US commercial healthcare claims databases for pharmacoepidemiology research
Source: BMC Med Res Methodol. 2023 Oct 21;23:246. doi: 10.1186/s12874-023-02073-6 (PMC10590518; doi:10.1186/s12874-023-02073-6)
Supplement: Supplementary file 1 — Supplementary Material 1 [file 12874_2023_2073_MOESM1_ESM.pdf]

Supplementary appendix to

Development and evaluation of an algorithm to link mothers and infants in two US commercial healthcare claims databases for pharmacoepidemiology research

James Weaver[1], MS, MPH

Jill H. Hardin[1], PhD

Clair Blacketer [1], MPH

Alexis A. Krumme[1], PhD

Melanie H. Jacobson[1], PhD

Patrick B. Ryan[1], PhD

[1]Janssen Research & Development. 1125 Trenton-Harbourton Rd, Titusville NJ 08560, USA

Contents:

A. Databases

B. Algorithm sensitivity implementation descriptions

C. Covariate construction

D. Person and record counts

E. Linkage algorithm sensitivity implementation 1 evaluation results

F. Linkage algorithm sensitivity implementation 2 evaluation results

G. References

A. Databases

Table S1. Database names and descriptions

Data source	Short name	Description
IBM MarketScan Commercial Claims and Encounters Database (CCAE)	CCAE	IBM MarketScan Commercial Claims and Encounters Database is a US employer-based private-payer administrative claims database. The data include adjudicated health insurance claims (e.g., inpatient, outpatient, and outpatient pharmacy) as well as enrollment data from large employers and health plans who provide private healthcare coverage to employees, their spouses, and dependents. Additionally, it captures laboratory tests for a subset of the covered lives. This administrative claims database includes a variety of fee-for-service, preferred provider organizations, and capitated health plans.
Optum® de-Identified Clinformatics® Data Mart Database – Date of Death (Clinformatics®)	Clinformatics®	Optum® De-Identified Clinformatics® Data Mart Database is an adjudicated administrative health claims database for members with private health insurance, who are fully insured in commercial plans or Medicare Advantage. The population is primarily representative of US commercial claims patients (0-65 years old) with some Medicare (65+ years old) however ages are capped at 90 years. It includes data captured from administrative claims processed from inpatient and outpatient medical services and prescriptions as dispensed, as well as results for outpatient lab tests processed by large national lab vendors who participate in data exchange with Optum. Optum DOD also provides date of death (month and year only) for members with both medical and pharmacy coverage from the Social Security Death Master File (however after 2011 reporting frequency changed due to changes in reporting requirements) and location information for patients is at the US state level.

B. Algorithm sensitivity implementation descriptions

The primary linkage algorithm implementation identified all births for a given mother and required ±60-day correspondence between the infant's date of birth (DOB) and the mother's pregnancy episode end date as described in Step 3. The first sensitivity analysis limited results to first births per mother only and maintained the ±60-day correspondence between the infant's DOB and the mother's pregnancy episode end. The second sensitivity analysis identified all births for a given mother and increased the correspondence between the infant's DOB and the mother's pregnancy episode end to ±90-days. The intent of limiting to first births was to assess whether linked and non-linked mother characteristic differences depended on multiparous status. The intent of extending the pregnancy end/DOB correspondence was to increase linkage sensitivity given that some day-of-birth values were inferred as 1 from typical insurance enrollment start dates and new children may not be enrolled with coverage immediately after birth.

C. Covariate construction

Mothers were characterized by age in years, index year (i.e., year in which pregnancy started/ended), index month, pre- and post-index database observation time in days, pregnancy episode length in days, and the Charlson Comorbidity Index (CCI)[1]. Infants were characterized by sex, index year, index month, and post-index observation time in days. Both mothers and infants were characterized by verbatim condition (SNOMED-CT) and aggregated condition group (SNOMED-CT clinical finding level) occurrences, drug ingredient level (Unified Medical Language System - RxNorm) and aggregated drug group (World Health Organization Anatomical Therapeutic Chemical Classification System 3rd, 4th, and 5th levels) exposures, procedure occurrences, measurement occurrence (but not values), and counts of distinct conditions, drug ingredient exposures, procedures, and measurements. Health care utilization was characterized by proxy using visit counts and counts of specific visit types (outpatient, inpatient, emergency department). Continuous measures were reported with mean, standard deviation, and median and clinical event occurrences were reported as proportions. We chose inferred DOB as the index date for linked infants to ensure covariate capture within enrollment periods where data are expected to be observed. We acknowledge the pregnancy episode end date better represents true birth date because live birth procedures are well-captured in claims data with reliable dates, whereas inferred DOB information is limited or redacted as part of enrollment and de-identification and enrollment date and is an imprecise birth marker. However, for calculating post-birth observation time, we set the index date as pregnancy episode end date.

D. Person and record counts

1) Linked mothers (pregnancy episode start)
   Mothers linked to ≥1 infant indexed at pregnancy episode start

2) Linked mothers (pregnancy episode end)
   Mothers linked to ≥1 infant indexed at pregnancy episode end

3) Linked infants
   Infants linked to a mother indexed at pregnancy episode end

4) Not linked mothers (pregnancy episode start)
   Mothers not linked to an infant indexed at pregnancy episode start

5) Not linked mothers (pregnancy episode end)
   Mothers not linked to an infant indexed at pregnancy episode end

6) Not linked infants
   Infants not linked to a mother indexed at inferred DOB

7) All candidate mothers (pregnancy episode start)
   Mothers without infant linkage restrictions indexed at pregnancy episode start

8) All candidate mothers (pregnancy episode end)
   Mothers without infant linkage restrictions indexed at pregnancy episode end

9) All candidate infants
   Infants without linkage restrictions indexed at DOB

Table S2. Person and record counts by database, algorithm implementation and cohort

Database	Algorithm implementation	Cohort Name	Records	Persons
CCAE	All pregnancies - 60d	Linked mothers (pregnancy episode start)	2,528,482	2,146,726
		Linked mothers (pregnancy episode end)	2,528,482	2,146,726
		Linked infants	2,528,482	2,528,482
		Not linked mothers (pregnancy episode start)	995,892	917,736
		Not linked mothers (pregnancy episode end)	995,892	917,736
		Not linked infants	2,406,894	2,406,894
		All mothers (pregnancy episode start)	3,608,039	3,064,263
		All mothers (pregnancy episode end)	3,608,039	3,064,263
		All infants	4,935,376	4,935,376
	First pregnancies - 60d	Linked mothers (pregnancy episode start)	2,146,698	2,146,698
		Linked mothers (pregnancy episode end)	2,146,698	2,146,698
		Linked infants	2,146,698	2,146,698
		Not linked mothers (pregnancy episode start)	917,764	917,764
		Not linked mothers (pregnancy episode end)	917,764	917,764
		Not linked infants	2,788,678	2,788,678
		All mothers (pregnancy episode start)	3,064,263	3,064,263
		All mothers (pregnancy episode end)	3,064,263	3,064,263
		All infants	4,935,376	4,935,376
	All pregnancies - 90d	Linked mothers (pregnancy episode start)	2,567,501	2,174,144
		Linked mothers (pregnancy episode end)	2,567,501	2,174,144
		Linked infants	2,567,501	2,567,499
		Not linked mothers (pregnancy episode start)	965,224	890,318
		Not linked mothers (pregnancy episode end)	965,224	890,318
		Not linked infants	2,367,877	2,367,877
		All mothers (pregnancy episode start)	3,608,039	3,064,263
		All mothers (pregnancy episode end)	3,608,039	3,064,263
		All infants	4,935,376	4,935,376
Clinformatics®	All pregnancies - 60d	Linked mothers (pregnancy episode start)	1,589,010	1,301,623
		Linked mothers (pregnancy episode end)	1,589,010	1,301,623
		Linked infants	1,589,010	1,589,010
		Not linked mothers (pregnancy episode start)	420,199	383,007
		Not linked mothers (pregnancy episode end)	420,199	383,007
		Not linked infants	1,790,801	1,790,801
		All mothers (pregnancy episode start)	1,986,803	1,684,615
		All mothers (pregnancy episode end)	1,986,803	1,684,615
		All infants	3,379,811	3,379,811
	First pregnancies - 60d	Linked mothers (pregnancy episode start)	1,219,859	1,219,859
		Linked mothers (pregnancy episode end)	1,219,859	1,219,859
		Linked infants	1,219,859	1,219,859
		Not linked mothers (pregnancy episode start)	464,771	464,771
		Not linked mothers (pregnancy episode end)	464,771	464,771
		Not linked infants	2,159,952	2,159,952
		All mothers (pregnancy episode start)	1,684,615	1,684,615
		All mothers (pregnancy episode end)	1,684,615	1,684,615
		All infants	3,379,811	3,379,811
	All pregnancies - 90d	Linked mothers (pregnancy episode start)	1,592,785	1,303,816
		Linked mothers (pregnancy episode end)	1,592,785	1,303,816
		Linked infants	1,592,785	1,592,785
		Not linked mothers (pregnancy episode start)	417,842	380,814
		Not linked mothers (pregnancy episode end)	417,842	380,814
		Not linked infants	1,787,026	1,787,026
		All mothers (pregnancy episode start)	1,986,803	1,684,615
		All mothers (pregnancy episode end)	1,986,803	1,684,615
		All infants	3,379,811	3,379,811

CCAE: IBM® Marketscan® Commercial Database; Clinformatics®: Optum's de-identified Clinformatics® Data Mart Database; Std.Diff.: Standardized difference of means

E. Linkage algorithm sensitivity implementation 1 evaluation results

Figure S1. Mother-infant linkage algorithm attrition diagram. Sensitivity algorithm implementation 1: first births, ±60-day pregnancy episode end/infant date-of-birth correspondence. Candidate persons: women whose pregnancy episode(s) ended with live birth and occurred during a mother's observation period; Candidate infants: persons who were 0 years of age at observation period start; Candidate links: candidate mothers-infants who matched on insurance enrollment ID infant's date-of-birth occurred during a candidate mother's observation period; Probable links: restricted candidate links to those where candidate infants date-of-birth occurred within ±60 days of the candidate mother's pregnancy episode end date; Inferred links: probable links where those where multiple mothers were associated with one infant were removed; CCAE: IBM® Marketscan® Commercial Database; Clinformatics®: Optum's de-identified Clinformatics® Data Mart Database

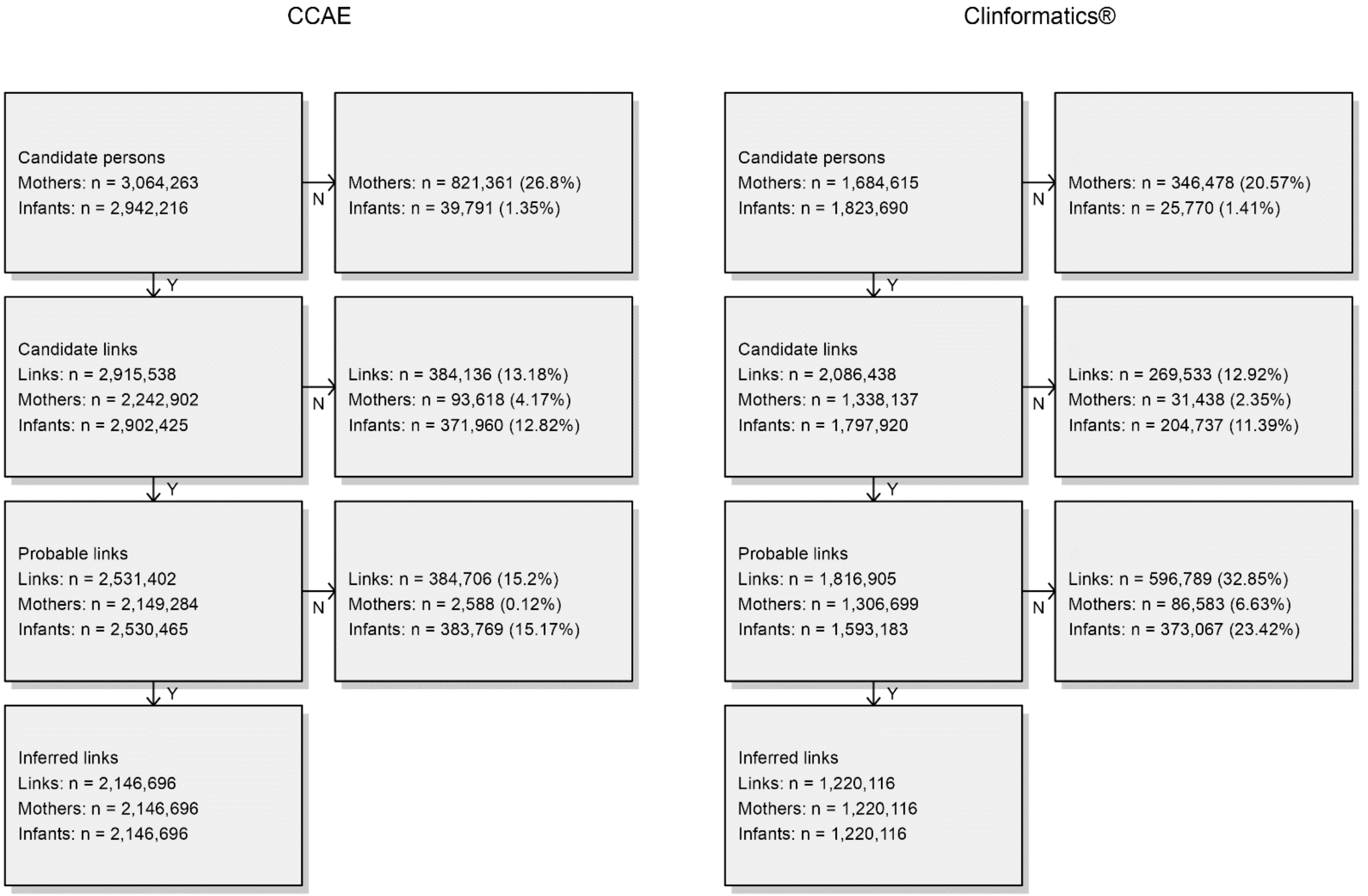
CCAE
Clinformatics®
Candidate persons
Mothers: n = 3,064,263
Infants: n = 2,942,216
Mothers: n = 821,361 (26.8%)
Infants: n = 39,791 (1.35%)
Candidate links
Links: n = 2,915,538
Mothers: n = 2,242,902
Infants: n = 2,902,425
Links: n = 384,136 (13.18%)
Mothers: n = 93,618 (4.17%)
Infants: n = 371,960 (12.82%)
Probable links
Links: n = 2,531,402
Mothers: n = 2,149,284
Infants: n = 2,530,465
Links: n = 384,706 (15.2%)
Mothers: n = 2,588 (0.12%)
Infants: n = 383,769 (15.17%)
Inferred links
Links: n = 2,146,696
Mothers: n = 2,146,696
Infants: n = 2,146,696
Candidate persons
Mothers: n = 1,684,615
Infants: n = 1,823,690
Mothers: n = 346,478 (20.57%)
Infants: n = 25,770 (1.41%)
Candidate links
Links: n = 2,086,438
Mothers: n = 1,338,137
Infants: n = 1,797,920
Links: n = 269,533 (12.92%)
Mothers: n = 31,438 (2.35%)
Infants: n = 204,737 (11.39%)
Probable links
Links: n = 1,816,905
Mothers: n = 1,306,699
Infants: n = 1,593,183
Links: n = 596,789 (32.85%)
Mothers: n = 86,583 (6.63%)
Infants: n = 373,067 (23.42%)
Inferred links
Links: n = 1,220,116
Mothers: n = 1,220,116
Infants: n = 1,220,116
Y
N

Figure S2. Scatter plots of demographic, drug exposure, condition, procedure, measurement, and visit occurrence covariate prevalence comparing linked vs. non-linked mother cohorts indexed at pregnancy episode start (row 1), linked vs non-linked mother cohorts indexed at pregnancy episode end (row 2). Sensitivity algorithm implementation 1: first births, ±60-day pregnancy episode end/infant date-of-birth correspondence. CCAE: IBM® Marketscan® Commercial Database; Clinformatics®: Optum's de-identified Clinformatics® Data Mart Database.

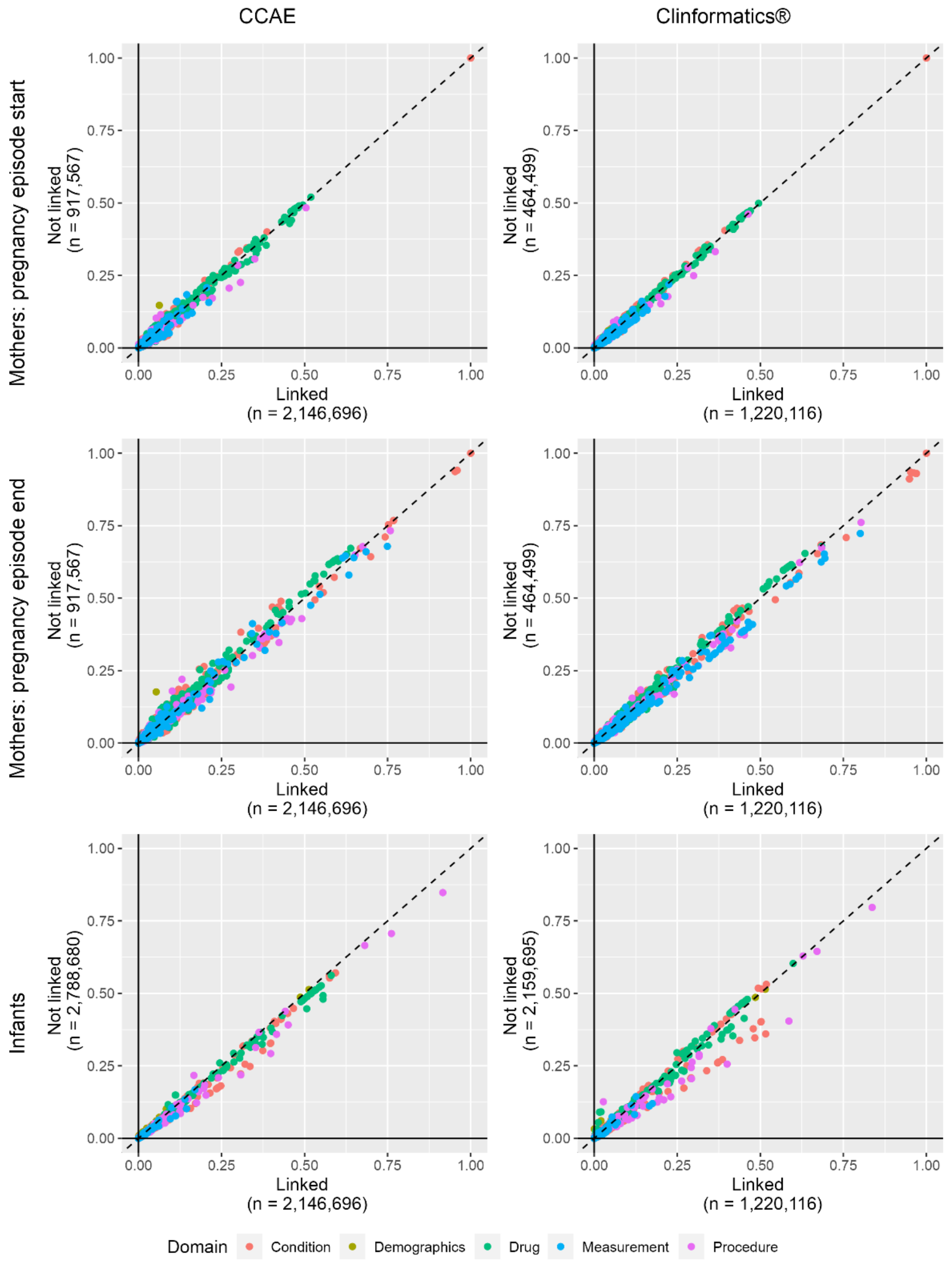

Table S3. Selected characteristics and standardized differences of linked and non-linked mothers in IBM® Marketscan® Commercial Database and Optum's de-identified Clinformatics® Data Mart Database. Mother characteristics were measured on the pregnancy episode start date (index year and month, age) or during the 365-day period before and including the pregnancy episode start date (distinct event occurrence counts). Sensitivity algorithm implementation 1: first births, ±60-day pregnancy episode end/infant date-of-birth correspondence.

	CCAE			Clinformatics®		
Characteristic	Linked % (n = 2,146,698)	Non-linked % (n = 917,764)	Std.Diff	Linked % (n = 1,219,859)	Non-linked % (n = 464,771)	Std.Diff
Index year						
2000	0.72	0.78	0.007	1.35	2.23	0.066
2001	1.08	1.21	0.013	4.01	5.53	0.071
2002	2.15	2.16	0	4.84	6.42	0.069
2003	3.46	2.77	-0.04	4.92	5.79	0.038
2004	4.34	3.53	-0.042	4.88	4.96	0.003
2005	4.16	3.37	-0.042	6.18	4.73	-0.064
2006	4.92	4.24	-0.033	6.31	5.28	-0.044
2007	5	4.47	-0.025	6.31	5.08	-0.053
2008	6.21	5.39	-0.035	5.85	4.75	-0.049
2009	6.28	5.98	-0.012	5.01	5.09	0.004
2010	7.6	6.62	-0.038	4.4	5.65	0.057
2011	8.24	8.49	0.009	4.21	5.94	0.079
2012	6.7	7.7	0.038	4.68	4.85	0.008
2013	6.78	7.18	0.016	4.59	4.32	-0.013
2014	5.68	6.67	0.041	4.32	4.19	-0.007
2015	5.05	5.77	0.032	4.56	4.36	-0.01
2016	4.66	5.21	0.025	4.69	4.43	-0.012
2017	4.62	4.9	0.013	4.85	4.47	-0.018
2018	4.91	5.08	0.008	4.89	4.22	-0.032
2019	4.24	4.59	0.017	4.57	3.96	-0.03
2020	3.19	3.9	0.038	4.45	3.63	-0.041
2021	0	0	0.007	0.12	0.14	0.007
Index month						
1	10.2	9.8	-0.013	9.1	9.19	0.003
2	7.73	10.88	0.109	7.87	8.26	0.014
3	6.26	14.69	0.278	7.94	8.96	0.037
4	7.49	8.42	0.034	7.45	7.63	0.007
5	8.5	7.01	-0.056	8.27	8.04	-0.008
6	8.36	6.72	-0.062	8.05	7.87	-0.007
7	8.8	6.81	-0.074	8.38	8.15	-0.008
8	8.69	6.76	-0.072	8.3	8.05	-0.009
9	8.6	7.02	-0.059	8.47	8.26	-0.008
10	8.65	7.35	-0.048	8.77	8.5	-0.01
11	8.41	7.16	-0.047	8.66	8.4	-0.009
12	8.32	7.39	-0.035	8.72	8.67	-0.002
Age (years)						
Mean	31.01	27.38	-0.449	30.64	28.37	-0.274
Std. deviation	4.74	6.52		4.95	6.63	
Median	31	27		31	28	
Prior observation time (days)						
Mean	590.75	750.23	0.146	581.03	655.96	0.075
Std. deviation	607.94	904.84		619.27	779.33	
Median	402	441		391	403	
Post observation time (days)						
Mean	1335.44	955.32	-0.254	1172.59	1021.04	-0.106
Std. deviation	1190.85	906.25		1062.44	950.33	
Median	908	643		784	686	

Pregnancy episode length (days)						
Mean	273.37	270.19	-0.107	273.1	271.46	-0.056
Std. deviation	18.5	23.19		18.77	22.26	
Median	279	277		278	279	
Distinct conditions						
Mean	6.3	6.33	0.003	6.76	6.83	0.006
Std. deviation	6.37	6.78		6.99	7.39	
Median	4	4		5	4	
Distinct drug ingredients						
Mean	4.57	4.48	-0.012	4.18	4.18	0.001
Std. deviation	5.41	5.43		5.18	5.31	
Median	3	3		3	3	
Distinct procedures						
Mean	7.13	6.3	-0.082	6.98	6.6	-0.036
Std. deviation	7.35	7.06		7.53	7.46	
Median	5	4		5	5	
Distinct measurements						
Mean	6.57	6.01	-0.033	13.79	11.6	-0.059
Std. deviation	12.03	11.75		27.65	25.14	
Median	3	2		3	3	
Distinct visit types						
Outpatient Visit						
Mean	6.28	5.3	-0.079	5.98	5.59	-0.031
Std. deviation	9.35	8.25		9.09	8.71	
Median	3	3		3	3	
Inpatient Visit						
Mean	0.08	0.08	0.001	0.07	0.08	0.005
Std. deviation	1.22	1.44		0.72	1.02	
Median	0	0		0	0	
Emergency Room Visit						
Mean	0.16	0.31	0.083	0.36	0.31	-0.007
Std. deviation	1.13	1.48		5.62	4.97	
Median	0	0		0	0	
Charlson comorbidity index						
Mean	0.17	0.18	0.012	0.2	0.21	0.012
Std. deviation	0.83	0.88		0.91	0.99	
Median	0	0		0	0	

CCAE: IBM® Marketscan® Commercial Database; Clinformatics®: Optum's de-identified Clinformatics® Data Mart Database; Std.Diff.: Standardized difference of means

Table S4. Selected characteristics and standardized differences of linked and non-linked mothers in IBM® Marketscan® Commercial Database and Optum's de-identified Clinformatics® Data Mart Database. Mother characteristics were measured on the pregnancy episode end date (index year and month, age) or during the 365-day period before and including the pregnancy episode end date (distinct event occurrence counts). Sensitivity algorithm implementation 1: first births, ±60-day pregnancy episode end/infant date-of-birth correspondence.

	CCAE			Clinformatics®		
Characteristic	Linked % (n = 2,146,698)	Non-linked % (n = 917,764)	Std.Diff	Linked % (n = 1,220,375)	Non-linked % (n = 464,255)	Std.Diff
Index year						
2000	0.11	0.33	0.047	0.01	0.02	0.012
2001	0.75	0.93	0.02	2.03	3.35	0.082
2002	1.31	1.61	0.025	4.47	6.15	0.075
2003	2.49	2.39	-0.006	4.93	6.43	0.065
2004	3.67	3	-0.037	4.88	5.51	0.029
2005	4.38	3.57	-0.041	5.12	4.53	-0.028
2006	4.22	3.48	-0.039	6.41	5.03	-0.059
2007	4.97	4.33	-0.03	6.31	5.29	-0.044
2008	5.35	4.84	-0.023	6.25	4.98	-0.055
2009	6.53	5.54	-0.042	5.69	4.67	-0.046
2010	6.27	6.43	0.007	4.69	5.45	0.035
2011	7.8	6.95	-0.033	4.32	5.74	0.065
2012	8.08	9.05	0.035	4.24	5.9	0.076
2013	6.52	6.91	0.016	4.89	4.45	-0.021
2014	6.79	7.3	0.02	4.45	4.25	-0.01
2015	5.21	5.97	0.033	4.41	4.25	-0.008
2016	5.14	5.8	0.029	4.71	4.45	-0.013
2017	4.66	5.02	0.017	4.7	4.45	-0.012
2018	4.68	4.98	0.014	4.86	4.41	-0.022
2019	4.77	4.93	0.007	4.87	4.18	-0.033
2020	4.16	4.48	0.016	4.47	3.83	-0.032
2021	2.14	2.15	0	3.29	2.67	-0.037
Index month						
1	8.17	6.66	-0.058	7.59	7.47	-0.005
2	7.79	6.14	-0.065	7.44	7.1	-0.013
3	8.68	6.76	-0.072	8.31	8.1	-0.008
4	8.48	6.56	-0.073	8.11	7.89	-0.008
5	8.86	6.81	-0.076	8.51	8.18	-0.012
6	8.58	7.12	-0.054	8.43	8.23	-0.007
7	8.64	7.39	-0.046	8.76	8.49	-0.009
8	8.66	7.39	-0.047	8.93	8.75	-0.006
9	8.44	7.55	-0.033	8.67	8.58	-0.003
10	9.81	9	-0.028	8.79	8.91	0.004
11	8.56	10.94	0.081	8.35	8.62	0.01
12	5.32	17.68	0.395	8.13	9.69	0.055
Age (years)						
Mean	31.77	27.99	-0.471	31.38	29.09	-0.278
Std. deviation	4.74	6.47		4.94	6.62	
Median	32	28		31	29	
Prior observation time (days)						
Mean	864.12	1020.43	0.143	854.17	927.43	0.074
Std. deviation	607.63	904.84		619.29	778.87	
Median	675	714		665	675	
Post observation time (days)						
Mean	1062.07	685.13	-0.252	899.9	748.3	-0.107
Std. deviation	1189.86	905.78		1061.97	947.24	

Median	635	372		512	414	
Distinct conditions						
Mean	20.01	20.6	0.039	21.28	20.79	-0.031
Std. deviation	10.2	11.26		10.75	11.35	
Median	18	18		19	18	
Distinct drug ingredients						
Mean	5.35	5.61	0.034	5.67	5.7	0.004
Std. deviation	5.38	5.6		5.42	5.68	
Median	4	4		4	4	
Distinct procedures						
Mean	17.72	16.8	-0.076	19.52	18.5	-0.081
Std. deviation	8.55	8.52		8.81	9.01	
Median	16	15		18	17	
Distinct measurements						
Mean	21.24	21.01	-0.01	46.41	40.93	-0.108
Std. deviation	15.77	16.67		36.8	35.2	
Median	18	18		36	27	
Distinct visit types						
Outpatient Visit						
Mean	15.46	14.23	-0.087	15.55	14.6	-0.062
Std. deviation	10.52	9.52		10.89	10.7	
Median	13	12		13	12	
Inpatient Visit						
Mean	1.13	1.13	0.002	1.13	1.12	-0.016
Std. deviation	0.63	0.69		0.55	0.62	
Median	1	1		1	1	
Emergency Room Visit						
Mean	0.34	0.65	0.139	1.03	0.9	-0.019
Std. deviation	1.32	1.81		5.01	4.64	
Median	0	0		0	0	
Charlson comorbidity index						
Mean	0.27	0.28	0.007	0.31	0.31	0.004
Std. deviation	0.89	0.94		0.97	1.05	
Median	0	0		0	0	

CCAE: IBM® Marketscan® Commercial Database; Clinformatics®: Optum's de-identified Clinformatics® Data Mart Database; Std.Diff.: Standardized difference of means

Table S5. Selected characteristics and standardized differences of linked and non-linked infants in IBM® Marketscan® Commercial Database and Optum's de-identified Clinformatics® Data Mart Database. Infant characteristics were measured on the pregnancy episode end date (index year and month, age, sex) or during the 365-day period after and including the pregnancy episode end date (distinct event occurrence counts). Sensitivity algorithm implementation 1: first births, ±60-day pregnancy episode end/infant date-of-birth correspondence.

	CCAE			Clinformatics®		
Characteristic	Linked % (n = 2,146,698)	Non-linked % (n = 2,788,678)	Std.Diff	Linked % (n = 1,220,375)	Non-linked % (n = 2,159,436)	Std.Diff
Sex						
gender = FEMALE	48.7	48.68	0	48.51	48.58	0.002
gender = MALE	51.3	51.32	0	51.49	51.42	-0.002
Index year						
2000	0.11	0.94	0.115	0.01	3.21	0.257
2001	0.75	0.93	0.02	2.03	6.05	0.205
2002	1.31	2.22	0.069	4.46	4.7	0.012
2003	2.49	3.5	0.059	4.93	4.62	-0.014
2004	3.67	3.89	0.012	4.88	4.48	-0.019
2005	4.38	4.17	-0.01	5.11	6.35	0.053
2006	4.22	4.72	0.024	6.41	6.09	-0.013
2007	4.97	4.68	-0.013	6.31	6.01	-0.012
2008	5.35	6.03	0.029	6.24	5.27	-0.042
2009	6.53	6.43	-0.004	5.7	4.48	-0.055
2010	6.27	7.25	0.039	4.69	3.83	-0.042
2011	7.8	7.28	-0.02	4.33	3.78	-0.028
2012	8.08	7.34	-0.028	4.23	3.75	-0.024
2013	6.52	6.51	0	4.89	4.28	-0.029
2014	6.8	6.52	-0.011	4.45	4.17	-0.013
2015	5.21	4.51	-0.032	4.42	4.68	0.013
2016	5.14	4.51	-0.03	4.72	4.83	0.005
2017	4.66	4.37	-0.014	4.7	4.67	-0.001
2018	4.68	4.71	0.002	4.86	4.46	-0.019
2019	4.77	3.98	-0.039	4.87	4.08	-0.038
2020	4.16	3.78	-0.02	4.47	3.56	-0.047
2021	2.15	1.75	-0.029	3.3	2.62	-0.04
Index month						
1	4.88	4.52	-0.017	7.45	6.35	-0.043
2	7.16	6.43	-0.029	7.4	6.16	-0.049
3	8.22	7.55	-0.025	8.29	7	-0.048
4	8.74	8.57	-0.006	8.11	7.19	-0.035
5	8.58	8.66	0.003	8.47	9.92	0.05
6	8.52	9.07	0.019	8.43	8.52	0.003
7	8.91	10.38	0.05	8.79	9.74	0.033
8	8.45	9.75	0.045	8.91	9.48	0.02
9	8.34	10.12	0.061	8.69	9.77	0.037
10	9.74	9.6	-0.005	8.8	9.27	0.016
11	9.47	7.96	-0.053	8.38	8.27	-0.004
12	8.98	7.38	-0.058	8.28	8.34	0.002
Post observation time (days)*						
Mean	1037.59	926.64	-0.067	808.32	794.99	-0.009
Std. deviation	1204.51	1131.93		1075.10	1025.66	
Median	596	515		394	422	
Distinct conditions						
Mean	6.79	5.89	-0.095	7.63	6.69	-0.09
Std. deviation	6.9	6.47		7.57	7.27	
Median	5	4		6	5	
Distinct drug ingredients						
Mean	2.66	2.51	-0.032	8.35	7.75	-0.036
Std. deviation	3.43	3.39		12.03	11.84	

Median	1	1		2	2	
Procedures						
Mean	11.54	10.11	-0.139	12.11	10.47	-0.151
Std. deviation	7.44	7.07		7.76	7.53	
Median	10	9		11	10	
Distinct measurements						
Mean	2.63	2.28	-0.05	3.26	2.93	-0.032
Std. deviation	5.02	4.88		7.42	7.58	
Median	1	1		1	1	
Distinct visit types						
Outpatient Visit						
Mean	10.05	8.87	-0.127	9.3	8.62	-0.063
Std. deviation	6.65	6.5		7.68	7.59	
Median	9	8		8	8	
Inpatient Visit						
Mean	0.68	0.52	-0.216	1.03	0.75	-0.411
Std. deviation	0.51	0.56		0.46	0.51	
Median	1	0		1	1	
Emergency Room Visit						
Mean	0.23	0.24	0.006	0.09	0.13	0.006
Std. deviation	0.85	0.9		3.87	3.56	
Median	0	0		0	0	

CCAE: IBM® Marketscan® Commercial Database; Clinformatics®: Optum's de-identified Clinformatics® Data Mart Database; Std.Diff.: Standardized difference of means

*Post-index observation time was measured from the linked mother's delivery date (true birth date) and not enrollment start date (inferred birth date)

F. Linkage algorithm sensitivity implementation 2 evaluation results

Figure S3. Mother-infant linkage algorithm attrition diagram. Sensitivity algorithm implementation 2: all births, ±90-day pregnancy episode end/infant date-of-birth correspondence. Candidate persons: women whose pregnancy episode(s) ended with live birth and occurred during a mother's observation period; Candidate infants: persons who were 0 years of age at observation period start; Candidate links: candidate mothers-infants who matched on insurance enrollment ID infant's date-of-birth occurred during a candidate mother's observation period; Probable links: restricted candidate links to those where candidate infants date-of-birth occurred within ±60 days of the candidate mother's pregnancy episode end date; Inferred links: probable links where those where multiple mothers were associated with one infant were removed; CCAE: IBM® Marketscan® Commercial Database; Clinformatics®: Optum's de-identified Clinformatics® Data Mart Database

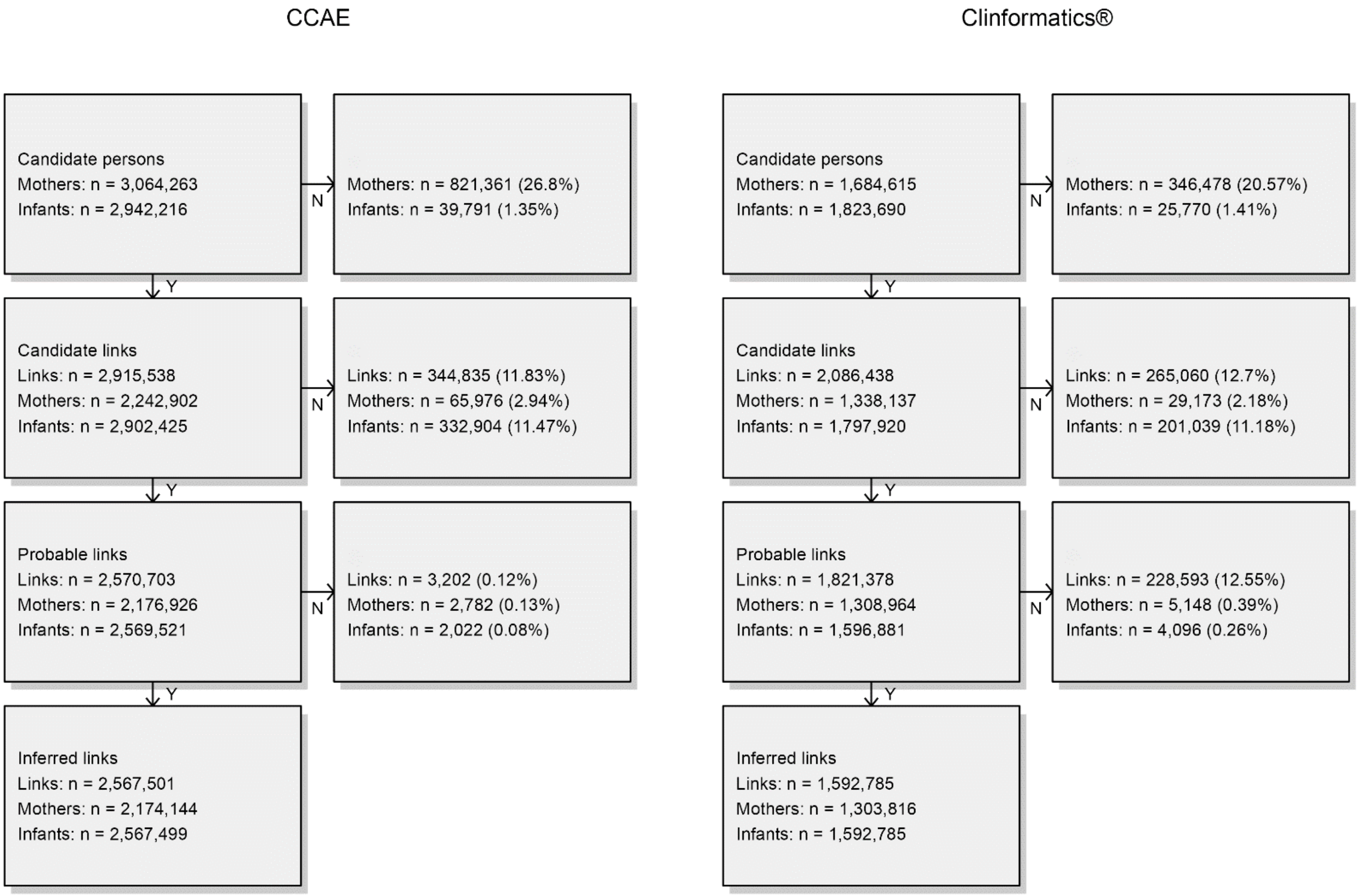
CCAE
Candidate persons
Mothers: n = 3,064,263
Infants: n = 2,942,216
N
Mothers: n = 821,361 (26.8%)
Infants: n = 39,791 (1.35%)
Y
Candidate links
Links: n = 2,915,538
Mothers: n = 2,242,902
Infants: n = 2,902,425
N
Links: n = 344,835 (11.83%)
Mothers: n = 65,976 (2.94%)
Infants: n = 332,904 (11.47%)
Y
Probable links
Links: n = 2,570,703
Mothers: n = 2,176,926
Infants: n = 2,569,521
N
Links: n = 3,202 (0.12%)
Mothers: n = 2,782 (0.13%)
Infants: n = 2,022 (0.08%)
Y
Inferred links
Links: n = 2,567,501
Mothers: n = 2,174,144
Infants: n = 2,567,499
Clinformatics®
Candidate persons
Mothers: n = 1,684,615
Infants: n = 1,823,690
N
Mothers: n = 346,478 (20.57%)
Infants: n = 25,770 (1.41%)
Y
Candidate links
Links: n = 2,086,438
Mothers: n = 1,338,137
Infants: n = 1,797,920
N
Links: n = 265,060 (12.7%)
Mothers: n = 29,173 (2.18%)
Infants: n = 201,039 (11.18%)
Y
Probable links
Links: n = 1,821,378
Mothers: n = 1,308,964
Infants: n = 1,596,881
N
Links: n = 228,593 (12.55%)
Mothers: n = 5,148 (0.39%)
Infants: n = 4,096 (0.26%)
Y
Inferred links
Links: n = 1,592,785
Mothers: n = 1,303,816
Infants: n = 1,592,785

Figure S4. Scatter plots of demographic, drug exposure, condition, procedure, measurement, and visit occurrence covariate prevalence comparing linked vs. non-linked mother cohorts indexed at pregnancy episode start (row 1), linked vs non-linked mother cohorts indexed at pregnancy episode end (row 2). Sensitivity algorithm implementation 2: all births, ±90-day pregnancy episode end/infant date-of-birth correspondence. CCAE: IBM® Marketscan® Commercial Database; Clinformatics®: Optum's de-identified Clinformatics® Data Mart Database.

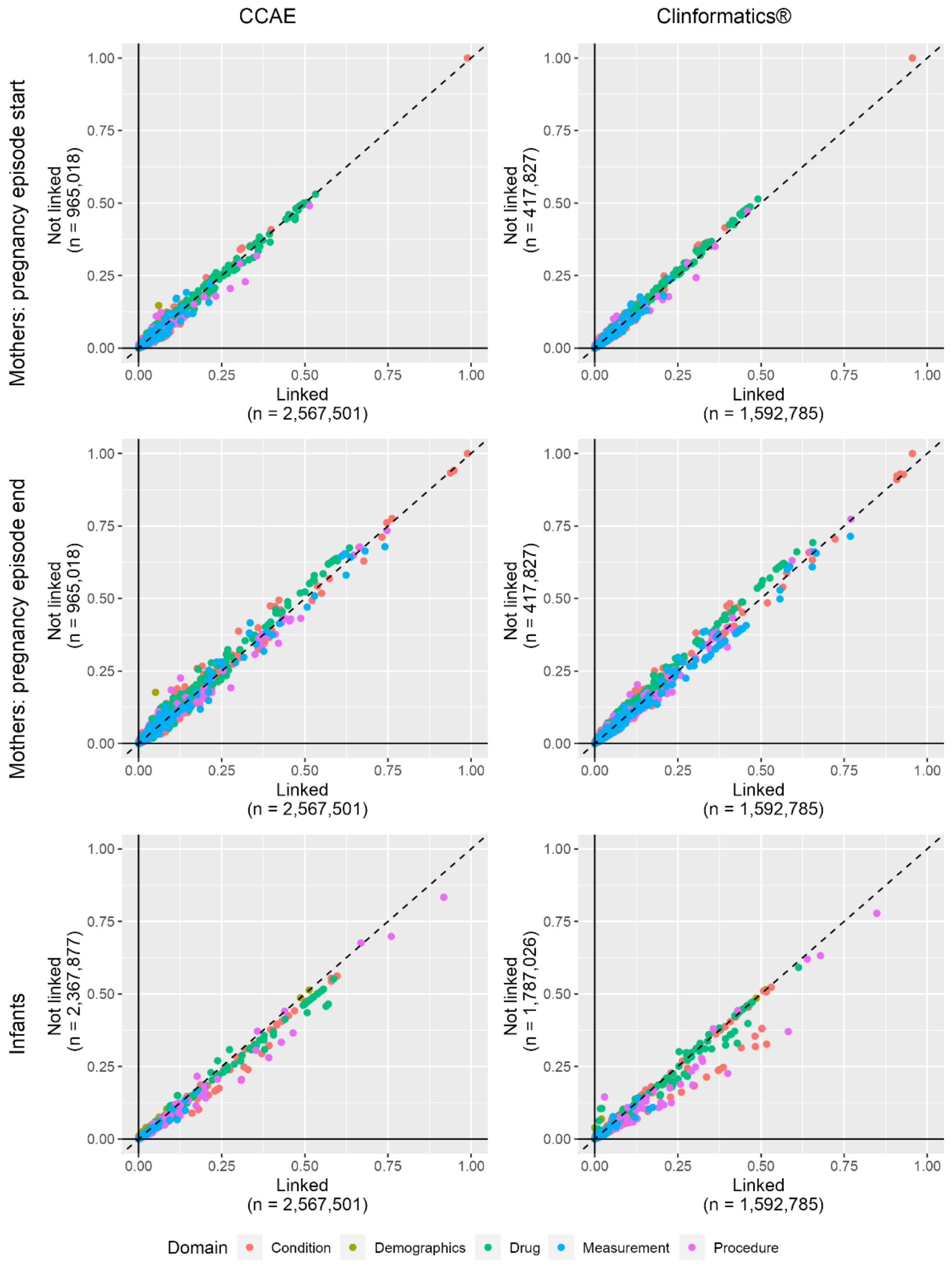

Table S6. Selected characteristics and standardized differences of linked and non-linked mothers in IBM® Marketscan® Commercial Database and Optum's de-identified Clinformatics® Data Mart Database. Mother characteristics were measured on the pregnancy episode start date (index year and month, age) or during the 365-day period before and including the pregnancy episode start date (distinct event occurrence counts). Sensitivity algorithm implementation 2: all births, ±90-day pregnancy episode end/infant date-of-birth correspondence.

	CCAE			Clinformatics®		
Characteristic	Linked % (n = 2,567,501)	Non-linked % (n = 965,224)	Std.Diff	Linked % (n = 1,592,785)	Non-linked % (n = 417,842)	Std.Diff
Index year						
2000	0.63	0.69	0.007	1.36	1.38	0.002
2001	0.97	1.05	0.008	3.84	3.76	-0.004
2002	1.95	1.97	0.001	4.72	4.53	-0.009
2003	3.15	2.54	-0.037	4.84	4.64	-0.009
2004	4.05	3.3	-0.04	4.8	4.48	-0.015
2005	3.99	3.19	-0.043	6.18	4.08	-0.095
2006	4.77	4.03	-0.036	6.3	4.76	-0.067
2007	4.95	4.3	-0.031	6.31	4.82	-0.065
2008	6.07	5.23	-0.036	5.92	4.64	-0.057
2009	6.18	5.82	-0.015	5.12	5.18	0.003
2010	7.43	6.48	-0.037	4.54	6	0.065
2011	8.13	8.42	0.011	4.38	6.46	0.092
2012	6.84	7.74	0.035	4.87	5.34	0.021
2013	6.92	7.35	0.016	4.71	4.82	0.005
2014	5.88	6.9	0.042	4.4	4.78	0.018
2015	5.34	6.04	0.03	4.59	5.03	0.02
2016	4.94	5.51	0.025	4.67	5.17	0.023
2017	4.85	5.16	0.015	4.8	5.28	0.022
2018	5.06	5.33	0.012	4.78	5.11	0.016
2019	4.49	4.82	0.016	4.44	4.89	0.022
2020	3.41	4.13	0.038	4.33	4.65	0.016
2021	0	0.01	0.008	0.11	0.18	0.017
Index month						
1	9.89	9.9	0	9.05	9.15	0.004
2	7.49	10.96	0.12	7.78	8.34	0.021
3	6.04	14.68	0.287	7.85	9.22	0.049
4	7.41	8.39	0.036	7.42	7.7	0.011
5	8.49	7.01	-0.055	8.24	7.96	-0.01
6	8.45	6.69	-0.066	8.08	7.79	-0.011
7	8.93	6.76	-0.081	8.42	8.09	-0.012
8	8.82	6.74	-0.078	8.37	8	-0.013
9	8.73	6.99	-0.065	8.52	8.18	-0.012
10	8.77	7.31	-0.054	8.79	8.47	-0.012
11	8.53	7.13	-0.052	8.7	8.4	-0.011
12	8.45	7.42	-0.038	8.79	8.7	-0.003
Age (years)						
Mean	31.22	27.25	-0.496	30.93	27.93	-0.357
Std. deviation	4.68	6.49		4.88	6.82	
Median	31	27		31	28	
Prior observation time (days)						
Mean	738.68	836.67	0.079	715.47	779.41	0.055
Std. deviation	744.33	985.54		741.19	894.21	
Median	505	487		476	473	
Post observation time (days)						
Mean	1361.93	941.77	-0.282	1221.32	929.25	-0.213
Std. deviation	1196.94	883.18		1092.65	828.35	
Median	932	640		825	641	

Pregnancy episode length (days)						
Mean	273.08	269.87	-0.107	272.69	270.43	-0.076
Std. deviation	18.59	23.46		18.99	23.11	
Median	278	277		278	278	
Distinct conditions						
Mean	6.67	6.79	0.012	6.94	7.45	0.046
Std. deviation	6.72	7.37		7.38	8.18	
Median	5	4		5	5	
Distinct drug ingredients						
Mean	4.68	4.62	-0.007	4.14	4.45	0.041
Std. deviation	5.39	5.5		5.17	5.5	
Median	3	3		3	3	
Distinct procedures						
Mean	7.39	6.61	-0.075	7.07	6.92	-0.014
Std. deviation	7.39	7.29		7.61	7.71	
Median	5	5		5	5	
Distinct measurements						
Mean	6.69	6.3	-0.023	13.61	12.48	-0.03
Std. deviation	11.9	11.9		27.48	25.89	
Median	3	3		3	3	
Distinct visit types						
Outpatient Visit						
Mean	6.61	5.49	-0.087	6.38	5.89	-0.035
Std. deviation	9.81	8.3		10.48	8.98	
Median	4	3		3	3	
Inpatient Visit						
Mean	0.11	0.11	-0.001	0.1	0.11	0.003
Std. deviation	1.11	1.27		0.77	0.94	
Median	0	0		0	0	
Emergency Room Visit						
Mean	0.16	0.34	0.092	0.38	0.33	-0.007
Std. deviation	1.17	1.56		6.15	4.89	
Median	0	0		0	0	
Charlson comorbidity index						
Mean	0.19	0.2	0.003	0.22	0.24	0.017
Std. deviation	0.85	0.89		0.93	1.02	
Median	0	0		0	0	

CCAE: IBM® Marketscan® Commercial Database; Clinformatics®: Optum's de-identified Clinformatics® Data Mart Database; Std.Diff.: Standardized difference of means

Table S7. Selected characteristics and standardized differences of linked and non-linked mothers in IBM® Marketscan® Commercial Database and Optum's de-identified Clinformatics® Data Mart Database. Mother characteristics were measured on the pregnancy episode end date (index year and month, age) or during the 365-day period before and including the pregnancy episode end date (distinct event occurrence counts). Sensitivity algorithm implementation 2: all births, ±90d pregnancy episode end/infant date-of-birth correspondence.

	CCAE			Clinformatics®		
Characteristic	Linked % (n = 2,567,501)	Non-linked % (n = 965,224)	Std.Diff	Linked % (n = 1,592,785)	Non-linked % (n = 417,842)	Std.Diff
Index year						
2000	0.1	0.31	0.048	0.01	0.02	0.01
2001	0.66	0.84	0.021	2.02	2.19	0.012
2002	1.18	1.43	0.022	4.27	4.28	0
2003	2.24	2.2	-0.002	4.82	4.66	-0.007
2004	3.35	2.77	-0.034	4.78	4.56	-0.011
2005	4.11	3.35	-0.04	5.07	4.04	-0.05
2006	4.05	3.29	-0.04	6.37	4.42	-0.086
2007	4.84	4.14	-0.034	6.3	4.84	-0.064
2008	5.28	4.67	-0.028	6.26	4.79	-0.064
2009	6.37	5.39	-0.042	5.8	4.59	-0.055
2010	6.18	6.26	0.003	4.8	5.63	0.037
2011	7.63	6.84	-0.031	4.49	6.14	0.073
2012	8.01	9.02	0.036	4.4	6.46	0.091
2013	6.7	7	0.012	5.07	4.91	-0.008
2014	6.93	7.48	0.021	4.55	4.75	0.009
2015	5.44	6.21	0.033	4.47	4.87	0.019
2016	5.43	6.07	0.027	4.73	5.12	0.018
2017	4.93	5.31	0.017	4.68	5.21	0.024
2018	4.89	5.24	0.016	4.8	5.23	0.02
2019	4.94	5.18	0.011	4.74	5.11	0.017
2020	4.42	4.71	0.014	4.34	4.77	0.021
2021	2.32	2.3	-0.001	3.23	3.44	0.012
Index month						
1	8.12	6.59	-0.059	7.56	7.42	-0.005
2	7.78	6.13	-0.065	7.43	7.04	-0.015
3	8.77	6.73	-0.076	8.33	7.99	-0.012
4	8.61	6.51	-0.08	8.15	7.81	-0.012
5	8.99	6.81	-0.081	8.58	8.12	-0.017
6	8.73	7.1	-0.06	8.47	8.19	-0.01
7	8.77	7.34	-0.052	8.77	8.5	-0.01
8	8.76	7.37	-0.051	8.96	8.74	-0.008
9	8.56	7.51	-0.039	8.72	8.62	-0.003
10	9.55	9.18	-0.013	8.75	8.87	0.004
11	8.25	11.09	0.096	8.26	8.67	0.015
12	5.11	17.64	0.403	8.03	10.02	0.07
Age (years)						
Mean	31.98	27.86	-0.519	31.67	28.65	-0.361
Std. deviation	4.68	6.43		4.88	6.81	
Median	32	27		32	28	
Prior observation time (days)						
Mean	1011.76	1106.54	0.077	988.16	1049.84	0.053
Std. deviation	744	985.32		740.95	894.03	
Median	778	756		749	744	
Post observation time (days)						
Mean	1088.85	671.9	-0.281	948.63	658.82	-0.212
Std. deviation	1195.92	882.7		1091.6	827.58	
Median	659	370		553	370	
Distinct conditions						
Mean	19.78	20.82	0.068	20.19	21.44	0.078

Std. deviation	10.21	11.43		10.65	11.96	
Median	17	18		18	19	
Distinct drug ingredients						
Mean	5.29	5.67	0.049	5.4	6	0.075
Std. deviation	5.34	5.63		5.39	5.84	
Median	4	4		4	4	
Distinct procedures						
Mean	17.44	16.84	-0.05	18.59	18.55	-0.003
Std. deviation	8.44	8.52		8.72	9.1	
Median	16	15		18	17	
Distinct measurements						
Mean	20.8	21.03	0.01	43.99	41.65	-0.046
Std. deviation	15.54	16.65		36.44	36.04	
Median	18	18		32	27	
Distinct visit types						
Outpatient Visit						
Mean	15.52	14.2	-0.089	15.7	14.76	-0.054
Std. deviation	11.33	9.52		13.45	10.96	
Median	13	12		12	12	
Inpatient Visit						
Mean	1.13	1.14	0.006	1.14	1.12	-0.018
Std. deviation	0.68	0.69		0.72	0.64	
Median	1	1		1	1	
Emergency Room Visit						
Mean	0.33	0.67	0.149	1.03	0.98	-0.007
Std. deviation	1.37	1.85		5.63	4.52	
Median	0	0		0	0	
Charlson comorbidity index						
Mean	0.3	0.3	0.001	0.32	0.34	0.016
Std. deviation	0.9	0.94		0.98	1.08	
Median	0	0		0	0	

CCAE: IBM® Marketscan® Commercial Database; Clinformatics®: Optum's de-identified Clinformatics® Data Mart Database; Std.Diff.: Standardized difference of means

Table S8. Selected characteristics and standardized differences of linked and non-linked infants in IBM® Marketscan® Commercial Database and Optum's de-identified Clinformatics® Data Mart Database. Infant characteristics were measured on the inferred pregnancy episode end date (index year and month, age) or during the 365-day period after and including the inferred pregnancy episode end date. (Distinct event occurrence counts). Sensitivity algorithm implementation 2: all births, ±90d pregnancy episode end/infant date-of-birth correspondence

	CCAE			Clinformatics®		
Characteristic	Linked<br>% (n = 2,567,501)	Non-linked<br>% (n = 2,367,877)	Std.Diff	Linked<br>% (n = 1,592,785)	Non-linked<br>% (n = 1,787,026)	Std.Diff
Sex						
gender = FEMALE	48.72	48.65	-0.002	48.58	48.55	-0.001
gender = MALE	51.28	51.35	0.002	51.42	51.45	0.001
Index year						
2000	0.09	1.1	0.131	0.01	3.88	0.283
2001	0.66	1.06	0.043	2.01	6.9	0.238
2002	1.18	2.52	0.1	4.27	4.93	0.031
2003	2.24	3.95	0.099	4.82	4.65	-0.008
2004	3.35	4.28	0.048	4.78	4.48	-0.014
2005	4.11	4.42	0.015	5.07	6.65	0.067
2006	4.05	4.98	0.045	6.37	6.07	-0.013
2007	4.84	4.77	-0.003	6.3	5.97	-0.014
2008	5.27	6.23	0.041	6.26	5.06	-0.052
2009	6.37	6.58	0.009	5.81	4.13	-0.077
2010	6.18	7.52	0.053	4.8	3.56	-0.062
2011	7.63	7.38	-0.01	4.5	3.51	-0.05
2012	8.01	7.27	-0.028	4.4	3.51	-0.046
2013	6.69	6.33	-0.015	5.07	3.99	-0.052
2014	6.94	6.32	-0.025	4.55	4.02	-0.026
2015	5.44	4.14	-0.061	4.47	4.68	0.01
2016	5.43	4.08	-0.064	4.73	4.84	0.005
2017	4.93	4.02	-0.044	4.68	4.68	0
2018	4.89	4.49	-0.019	4.8	4.44	-0.017
2019	4.94	3.66	-0.063	4.74	4.04	-0.034
2020	4.42	3.43	-0.051	4.33	3.49	-0.044
2021	2.33	1.49	-0.061	3.24	2.54	-0.042
Index month						
1	4.72	4.63	-0.004	7.41	6.16	-0.05
2	7.01	6.46	-0.022	7.38	5.92	-0.059
3	8.13	7.53	-0.022	8.29	6.73	-0.059
4	8.82	8.45	-0.013	8.15	6.96	-0.045
5	8.68	8.57	-0.004	8.54	10.16	0.056
6	8.66	9.02	0.012	8.48	8.49	0.001
7	9.05	10.49	0.049	8.8	9.93	0.039
8	8.55	9.87	0.045	8.94	9.57	0.022
9	8.46	10.3	0.063	8.74	9.95	0.041
10	9.71	9.62	-0.003	8.78	9.38	0.021
11	9.33	7.84	-0.054	8.3	8.32	0.001
12	8.87	7.22	-0.061	8.2	8.43	0.009
Post observation time (days)*						
Mean	1064.77	879.59	-0.113	855.36	750.73	-0.07
Std. deviation	1210.01	1107.74		1103.43	985.22	
Median	621	484		432	395	
Distinct conditions						
Mean	6.78	5.74	-0.11	7.75	6.39	-0.13
Std. deviation	6.89	6.4		7.65	7.1	
Median	5	4		6	5	
Distinct drug ingredients						
Mean	2.69	2.45	-0.05	8.55	7.44	-0.066
Std. deviation	3.43	3.38		12.05	11.76	

Median	1	1		2	2	
Procedures						
Mean	11.5	9.9	-0.157	12.27	9.99	-0.212
Std. deviation	7.42	7.01		7.83	7.36	
Median	10	9		11	9	
Distinct measurements						
Mean	2.61	2.24	-0.054	3.29	2.83	-0.044
Std. deviation	4.96	4.93		7.43	7.61	
Median	1	1		1	1	
Distinct visit types						
Outpatient Visit						
Mean	10.06	8.66	-0.151	9.47	8.32	-0.107
Std. deviation	6.65	6.47		7.74	7.5	
Median	9	8		9	7	
Inpatient Visit						
Mean	0.67	0.5	-0.217	1.03	0.69	-0.491
Std. deviation	0.51	0.57		0.47	0.51	
Median	1	0		1	1	
Emergency Room Visit						
Mean	0.22	0.24	0.016	0.1	0.13	0.006
Std. deviation	0.84	0.91		3.94	3.45	
Median	0	0		0	0	

CCAE: IBM® Marketscan® Commercial Database; Clinformatics®: Optum's de-identified Clinformatics® Data Mart Database; Std.Diff.: Standardized difference of means

*Post-index observation time was measured from the linked mother's delivery date (true birth date) and not enrollment start date (inferred birth date)

G. References

1. Charlson, M.E., et al., *A new method of classifying prognostic comorbidity in longitudinal studies: development and validation.* J Chronic Dis, 1987. **40**(5): p. 373-83.
